# Microfluidics and molecular diagnostics in renal cell carcinoma: advances, challenges, and future directions

**DOI:** 10.3389/fonc.2025.1674789

**Published:** 2025-12-17

**Authors:** Sadeq B. Abu-Dawas, Aiman Y. Alwadi, Yara H. Farahat, Reema B. Abu-Dawas, Lama N. Quraiba, Reem A. Abu-Omar, Khaled AlKattan, Mohammed Imran Khan, Ahmed Yaqinuddin

**Affiliations:** 1 College of Medicine, Alfaisal University, Riyadh, Saudi Arabia; 2 Department of Pharmaceutical Sciences, College of Pharmacy, Alfaisal University, Riyadh, Saudi Arabia; 3 Research Centre, King Faisal Specialist Hospital & Research Centre, Jeddah, Saudi Arabia

**Keywords:** renal cell carcinoma (RCC), microfluidics, molecular diagnostics, liquid biopsy, biomarkers, precision oncology

## Abstract

RCC represents the predominant form of kidney cancer, with rising global incidence and notable mortality despite advancements in diagnosis and treatment. Traditional imaging and histopathological techniques, while foundational, face limitations in early detection, subtype differentiation, and treatment personalization. This review comprehensively explores RCC’s clinical and pathological landscape, then transitions to focus on recent innovations in microfluidics and molecular diagnostics that are reshaping kidney cancer management. Microfluidic platforms facilitate efficient, minimally invasive analysis of biomarkers such as CTCs, ctDNA, and exosomes, enabling real-time disease monitoring and drug response assessment. Biomolecular technologies—including single-cell sequencing, spatial transcriptomics, and next-generation sequencing—offer deeper insights into tumor heterogeneity and therapeutic resistance. The integration of multi-omics data and emerging platforms like kidney cancer-on-a-chip highlight the promise of precision medicine. Challenges in clinical translation, including assay standardization and regulatory hurdles, are also addressed. Together, these developments underscore a paradigm shift toward individualized, biomarker-driven care in RCC.

## 1 Introduction

Kidney cancer is a significant global health condition with an estimation of 434,840 new cases and 155,953 related deaths reported in 2022 (1). Renal Cell Carcinoma (RCC) represents about 90% of all kidney cancer diagnoses; remaining 10% include transitional cell carcinoma, Wilms tumor, and other rarer types (2). The global incidence of RCC continues to rise, particularly in high-income countries, largely due to increasing prevalence of modifiable risk factors and expanded use of imaging for early detection, while age-standardized mortality rates have plateaued in several developed regions (3–5). The highest incidence rates are reported in the regions of North America and Northern Europe, exceeding 15 cases annually per 100,000 population, whereas the lowest are observed in Africa and Southeast Asia (4, 6). Despite advancements in diagnostic techniques and therapeutic strategies, mortality associated with RCC remains substantial. Globally, RCC is still among the ten leading causes of cancer-related mortality in men (2). There were very large variations between countries in RCC mortality rates, often exceeding what would be expected based on incidence rates alone, thus underscoring disparities in access to healthcare, early diagnosis, and treatment facilities. Epidemiologically, RCC occurs about twice as often in males as in females (7). It is a disease mostly affecting young adults nearly between the age of 50 and 70, with a median age at diagnosis of 64 years in the United States (7). Early-stage tumors are increasingly being detected by imaging largely as incidental findings; however, late-stage diagnoses still predominate due to the limited access to diagnostic services in many of these areas (3). These pressing concerns continue to call for more efficient diagnostic platforms such as microfluidic and biomolecular technologies to make early detections possible and improve clinical outcomes.

**Figure 1:**
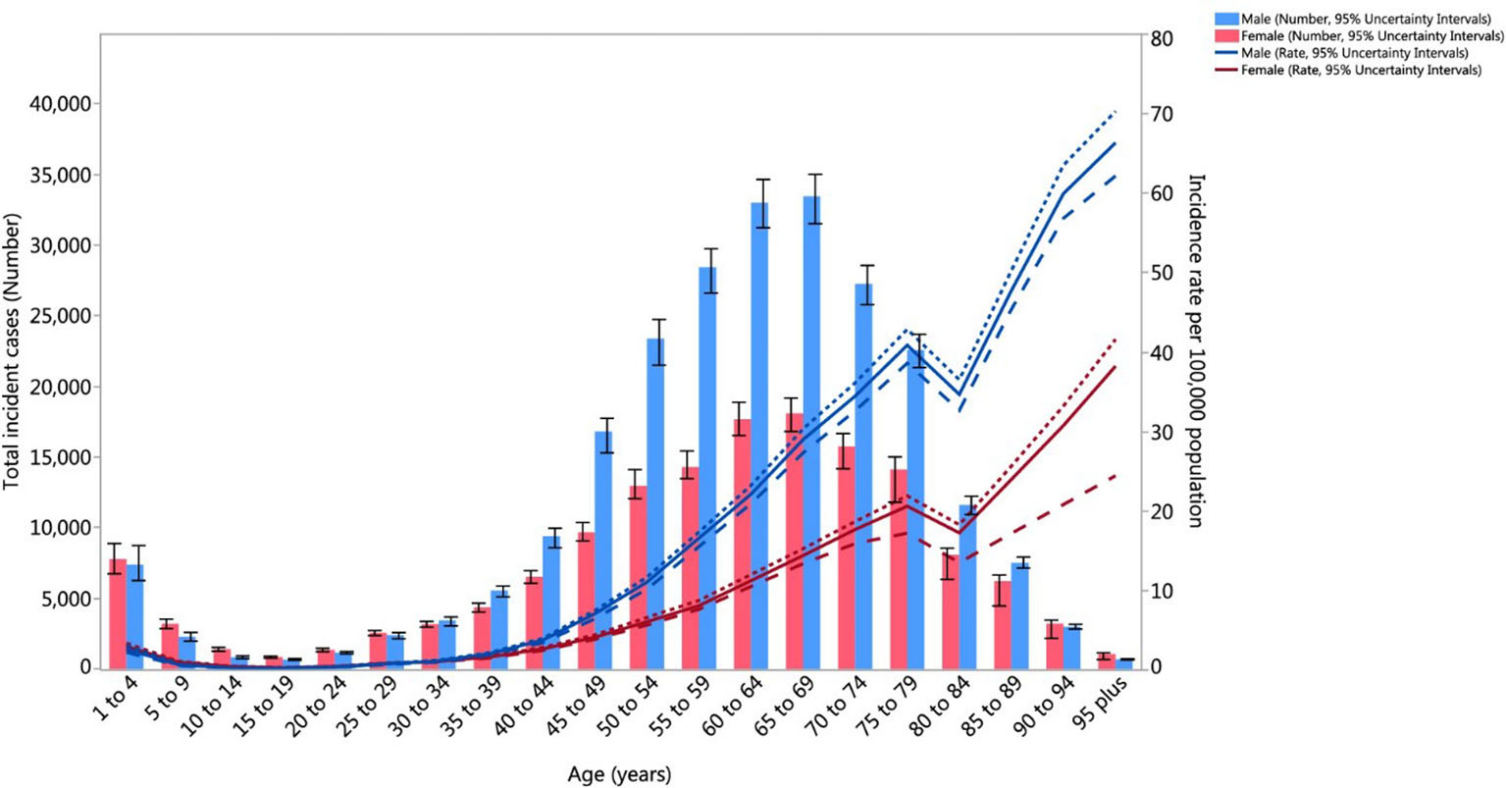
Age-Standardized Global Incidence of Kidney Cancer per 100,000 population by age and sex, 2017. Incidence rates increase markedly with age, peaking between 65–69 years, where males exhibit the highest burden (Reproduced from (8), under CC BY 4.0 license).

**Figure 1** illustrates distinct age-related patterns in kidney cancer incidence between males and females worldwide. Males aged 65 to 69 show the highest risk of developing kidney cancer and the incidence increases more rapidly in men than in women starting at age 40 (8). The pattern observed points to the role of smoking habits and occupational exposure as critical biological factors in kidney cancer. The total number of kidney cancer cases in older adults drops, however, the incidence rates rise due to a decreasing male survivor population. The narrow 95% uncertainty intervals represented by dashed and dotted lines shows that these trends are measured reliably (8).

The etiology of kidney cancer involves a combination of modifiable and non-modifiable risk factors. Lifestyle-related risk factors such as smoking, obesity, and hypertension increase the likelihood of developing RCC. Evidence indicates that smoking not only increases the likelihood of RCC but also correlates with reduced survival outcomes (9). Even though obesity is stated to be a risk, it has paradoxically been observed that overweight and obese patients survive better (10). Meanwhile, hypertension generally stands out as one of the most important risk factors of RCCs (9).

Nonmodifiable risk factors for RCC are genetic predisposition and family history (11). Inherited cancers, or cancer syndromes, are responsible for about 5% of kidney cancer cases (12). If a person has a first-degree relative with kidney cancer, his/her risk of developing RCC is very high (11). A person who inherits a pathogenic variant that causes hereditary RCC has a 50% lifetime risk of developing the disease (13). Preventive strategies note modifiable factors and focus on lifestyle approaches such as smoking cessation, weight management through a healthy diet, and effective control of hypertension (14). Public health initiatives for risk reduction and targeted screening programs for high-risk individuals can be instrumental in encouraging early detection and better outcomes for RCC (15).

**Figure 2:**
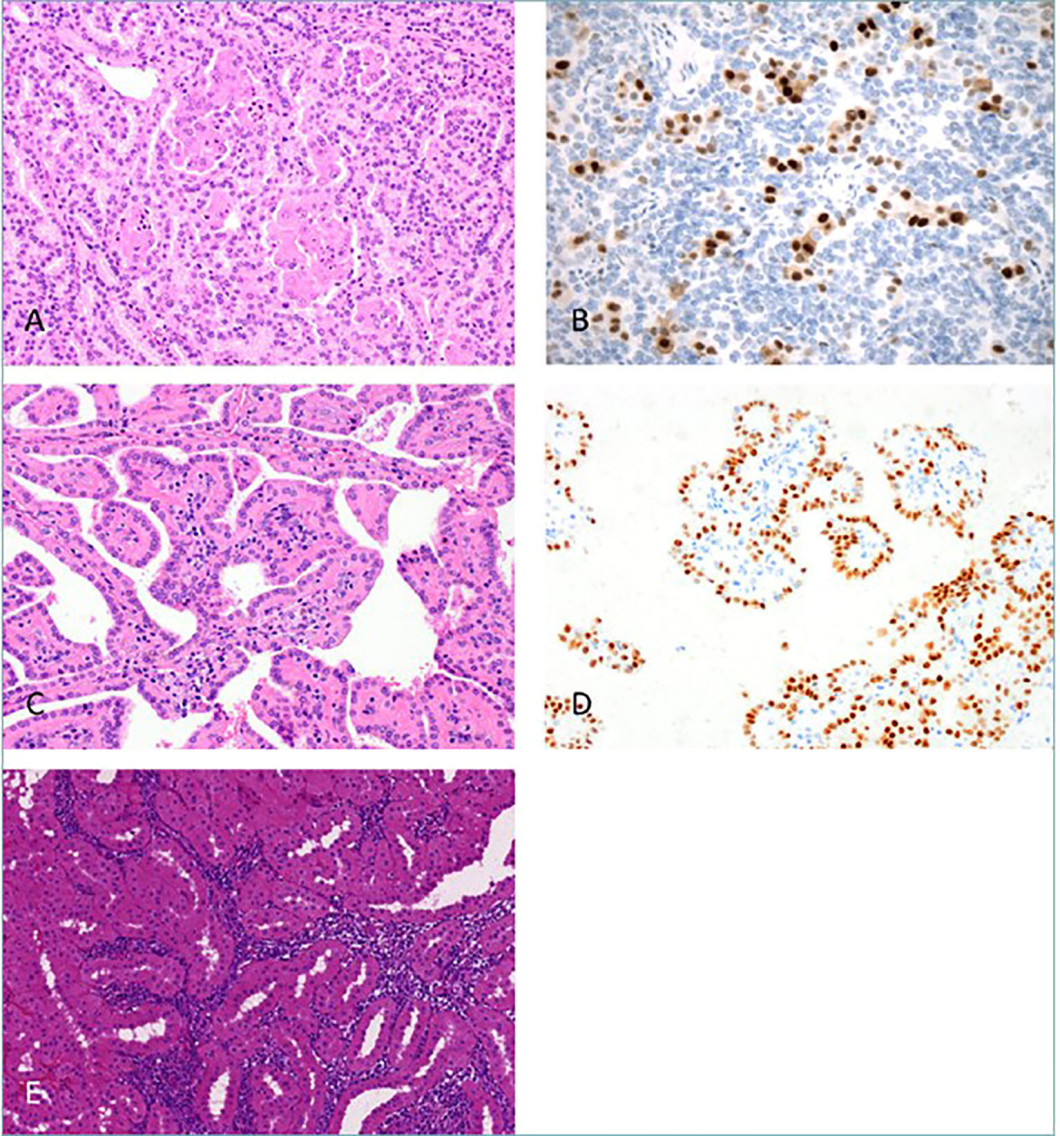
New Papillary renal cell carcinoma (pRCC) patterns. The panel illustrates novel histological patterns of pRCC as per the WHO 2022 classification. **(A)** Biphasic pRCC with emperipolesis; **(B)** Cyclin-D1 positive larger eosinophilic cells; **(C)** pRCC with reverse nuclear polarity; **(D)** GATA3 highlights reverse polarity; **(E)** Warthin-like pRCC with dense inflammation (Reproduced from (17), under CC BY 4.0 license).

The 2022 classification of World Health Organization (WHO) is an important step forward to better classify and study renal tumors. This classification integrates histological architecture with molecular and genetic features to define tumor subtypes (16). Renal tumors have been classified into standard categories including clear cell, papillary, oncocytic/chromophobe, collecting duct, and molecularly defined tumors, among others (17). This new system of classification also integrates immunohistochemistry (IHC) and DNA-based analyses to supplement the traditional morphological approach to define new tumor entities. Noteworthy here is that the previously used subclassification of papillary renal cell carcinomas (pRCC) into type 1 and type 2 is no longer accepted; now pRCC in the current classification is understood as a heterogeneous group with recognized variants such as biphasic pRCC and Warthin-like pRCC (17; see **Figure 2**).

Additional diagnostic categories were added to the 2022 revision of the RCC classification to give a finer representation of the genetic architecture underlying these tumors. Most importantly, eosinophilic solid and cystic RCC, ALK-rearranged RCC, and ELOC-mutated RCC were now accepted as entities based on validated genetic and IHC criteria (17, 18). The very term “molecularly defined renal carcinomas” emphasizes how critical the relation between genetic abnormality and tumor phenotype is, so that subtypes can be correlated with particular molecular abnormalities (17). This enhances diagnostic accuracy and then moves toward the consideration of targeted therapeutic strategies. For example, findings from BRAF mutation assays emphasize the clinical relevance of elucidating genetic drivers in RCC to inform prognosis and guide targeted interventions (19).

Histopathological diagnosis in pRCC is nevertheless troubled by limited ability to detect pRCC at early stages and to discriminate accurately among its subtypes (20). pRCC lesions at early stages tend to be small and barely perceptible making them difficult to be recognized through routine microscopic evaluation (21). Some pRCC subtypes, for example Warthin-like pRCC, may favorably mimic benign lesions, especially in the presence of background inflammation and nuclear atypia, thereby further complicating diagnosis (22). These diagnostic uncertainties prompt the need for some ancillary diagnostic modalities, such as IHC stains and molecular assays, so as to make the diagnosis more certain. Diagnosing solely by histological morphology will give rise to misclassification and hence suboptimal management of patients (23–25).

By manipulating tiny fluid volumes within microscale devices, the term “microfluidics” has given a new dimension toward the diagnosis and management of kidney cancer (26). These platforms enable the efficient isolation and analysis of biomarkers that occur in very low abundance, such as circulating tumor cells (CTC), cell-free DNA, and exosomes, to facilitate a fast-time, non-invasive diagnosis (27). Microfluidic systems are known to use much less reagents and are much faster in processing than traditional methods. It has been demonstrated that inertial microfluidics and a size-based filtration method capture viable kidney cancer cells for downstream analysis (28, 29). Furthermore, design for therapeutics-validation methods based on microfluidics integrated with biomolecular devices is increasingly being applied for controlled physiological modeling. This parametric system enables the real-time monitoring of mutation profiles and drug responses specific to individual patients, thus allowing for patient-specific treatment regimens (30). It is with the support of microfluidic platforms merged with molecular diagnostics that researchers expect to indeed advance early tumor detection and overcome therapeutic resistance in kidney cancer.

This review aims to provide a comprehensive overview of RCC, covering its epidemiology, risk factors, clinical presentation, and diagnostic challenges. It evaluates current diagnostic and therapeutic approaches while highlighting their limitations in early detection and personalized treatment. The review emphasizes recent advancements in microfluidics and molecular diagnostics, exploring their role in improving biomarker detection, disease monitoring, and therapy optimization. Additionally, it discusses emerging biomolecular technologies such as single-cell sequencing, liquid biopsy, and multi-omics integration, underscoring their potential in enhancing precision oncology. Finally, the review outlines future directions for the clinical translation of these innovations in kidney cancer management.

## 2 Kidney cancer: diagnosis and clinical management

### 2.1 Clinical presentation and symptoms

#### 2.1.1 Common symptoms

RCC is the most frequent kidney cancer, often detected incidentally due to increased use of imaging, with over 60% of cases discovered by chance (31). Classic symptoms such as hematuria, flank pain, and palpable abdominal mass are now less frequent, occurring in 4-7% of patients (32). Only about 30% of patients develop noticeable symptoms, leading to metastasis in 20-30% of cases (32).

Other possible symptoms include abdominal pain, fever, weight loss, fatigue from anemia, bone pain, or breathing difficulties in advanced stages. Paraneoplastic syndromes may cause hypertension, hypercalcemia, or liver-related issues. Physical examination may suggest RCC through findings such as abdominal mass, limb swelling, or varicocele due to renal vein thrombosis, but cannot definitively diagnose the disease.

#### 2.1.2 Paraneoplastic syndromes associated with kidney cancer

Up to 40% of RCC patients develop paraneoplastic syndromes (33). Hypercalcemia, often caused by tumor-derived PTHrP (parathyroid hormone–related protein), leads to systemic symptoms (34). Polycythemia due to erythropoietin secretion and Stauffer syndrome, marked by hepatic dysfunction, are also observed (35). Cachexia and fever result from cytokine release and elevated VEGF or renin levels (36).

#### 2.1.3 Asymptomatic presentation and incidental detection

Using ultrasound, computed tomography (CT), and magnetic resonance imaging (MRI) techniques has made it likely that more people with totally asymptomatic renal tumors will be found (37). Although early detection improves management, survival benefits remain uncertain. Doctors may not immediately treat certain lesions found by accident, usually called incidentalomas. In cases where the tumor is under 3 cm or if the patient has serious other medical problems, active surveillance is frequently recommended (38). Nephron-sparing surgery remains the standard for localized tumors, with ablation therapies as alternatives for non-surgical candidates.

### 2.2 Diagnostic methods

RCC is diagnosed by using different imaging methods together with microscopic analysis of tissue samples (39). As RCC initially shows no symptoms, tests with various diagnostic methods should be used before the condition develops further. A combination of clear pictures with purposeful biopsies supports the accurate detection of RCC (40). New biomarkers make it possible to more accurately identify whether a lesion is cancerous or not.

#### 2.2.1 Imaging techniques

Accurate imaging plays a central role in the diagnosis, staging, and treatment planning of RCC (**Table 1**). CT and MRI remain the most important tests for distinguishing malignant from benign renal tumors and for evaluating disease extent. Contrast-enhanced CT is the current gold standard for RCC diagnosis and staging because it provides high-resolution anatomic detail and clear enhancement patterns that allow differentiation between malignant and benign lesions (32, 38). Multiphasic CT protocols comprising unenhanced, corticomedullary, nephrographic, and excretory phases permit assessment of vascular characteristics and tumor perfusion, which aid in subtyping and surgical planning (41). CT also enables accurate detection of perinephric extension, venous invasion, lymph-node enlargement, and distant metastases. However, its use is limited by ionizing radiation and the risk of contrast-induced nephropathy, especially in patients with renal impairment (32, 41).

**Table 1:** Overview of imaging modalities in renal cell carcinoma.

Modality	Key diagnostic role	Advantages	Limitations/remarks
Ultrasound	Initial evaluation; distinguishes cystic vs. solid lesions	Widely available; no radiation	Limited for small/isoechoic lesions; operator dependent
Contrast-Enhanced Ultrasound	Real-time perfusion and vascular assessment	Safe in renal impairment; no nephrotoxic contrast	Limited depth; availability varies
Computed Tomography	Gold standard for diagnosis, staging, and surgical planning	High spatial resolution; evaluates vasculature and metastases	Radiation exposure; contrast nephropathy risk
Magnetic Resonance Imaging	Soft-tissue contrast; venous tumor-thrombus evaluation	No radiation; DCE/DWI sequences for characterization	Higher cost; longer scan time; limited access
Positron Emission Tomography	Functional metabolic imaging; detects metastasis and recurrence	Whole-body coverage; CAIX/PSMA tracers improve sensitivity	FDG less sensitive for primary RCC; tracer excretion interference

**Figure 3:**
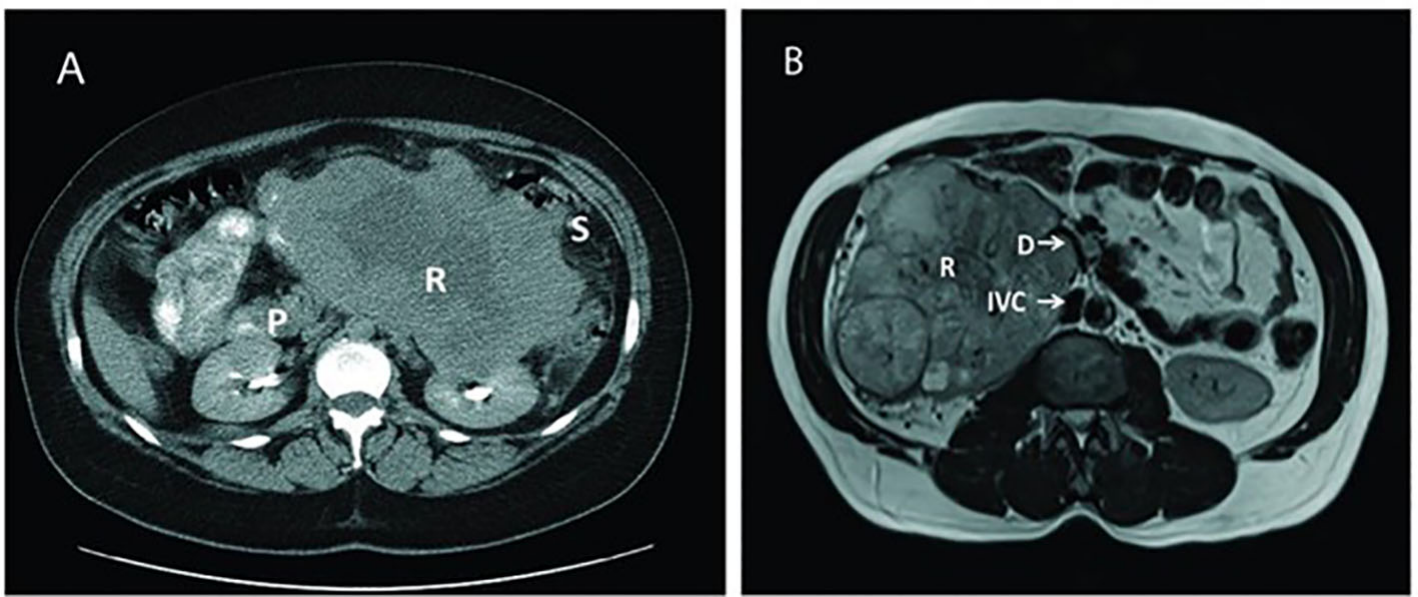
Computed tomography (CT) and magnetic resonance imaging (MRI) of two different patients with large renal mass. In the CT scan **(A)**, the left kidney tumor shows the blurring of soft tissue boundaries around the pancreas, spleen, and splenic flexure, indicating it may have invaded the surrounding organs. Alternatively, imaging with MRI **(B)** confirms a large, mixed solid-cystic tumor on the right side of the kidney pushing on and narrowing the nearby duodenum. P: Pancreas; R: Renal Tumor; S: Splenic flexure of colon; IVC: Inferior Vena Cava: D: Duodenum (Reproduced from (42), under CC BY 4.0 license).

MRI provides superior soft-tissue contrast and serves as a valuable alternative when iodinated contrast is contraindicated. It is particularly useful for evaluating venous tumor thrombus in the renal vein or inferior vena cava, as well as for characterizing indeterminate lesions detected on CT (38). Dynamic contrast-enhanced (DCE) and diffusion-weighted imaging (DWI) sequences add functional information that helps distinguish tumor subtypes and assess aggressiveness (38). **Figure 3** illustrates imaging techniques using MRI and CT for two patients, demonstrating enhanced renal tumor visualization and the added value of combining both modalities when assessing large or complex masses. MRI is also preferred for differentiating fat-poor angiomyolipoma, oncocytoma, and RCC subtypes when CT findings are inconclusive (42). Emerging metabolic imaging techniques, such as hyperpolarized ^13^C MRI, show potential for future application by allowing real-time visualization of tumor metabolism and improving early detection and functional assessment of RCC (43, 44).

Ultrasound remains the first-line imaging modality for initial renal-mass evaluation owing to its accessibility, absence of radiation, and ability to distinguish simple cysts from solid lesions. However, routine grayscale ultrasound has limited sensitivity for small or isoechoic tumors and cannot reliably distinguish benign from malignant solid masses (45). Contrast-enhanced ultrasound (CEUS) has expanded the diagnostic role of ultrasound by using intravascular microbubble contrast agents that provide real-time assessment of lesion vascularity and perfusion without nephrotoxic effects (45). Malignant lesions such as RCC typically exhibit rapid arterial enhancement followed by early washout, whereas benign lesions like oncocytoma and angiomyolipoma show slower, persistent enhancement. CEUS is particularly beneficial for patients with renal insufficiency or contrast allergy and can be used when CT or MRI contrast is contraindicated (45).

Positron emission tomography combined with computed tomography (PET/CT) also contributes to RCC evaluation. Although ^18^F-fluorodeoxyglucose PET/CT is widely used in oncology, its sensitivity for primary RCC is limited by variable glucose metabolism and physiologic tracer excretion through the kidneys (46). Nonetheless, recent studies have demonstrated improved performance using novel tracers that target RCC-specific biomarkers such as carbonic anhydrase IX (CAIX) and prostate-specific membrane antigen (PSMA). These radiotracers enhance detection of metastatic and recurrent disease and provide valuable information on treatment response in patients receiving targeted or immune therapy (46).

#### 2.2.2 Biopsy and histopathological analysis

Renal mass biopsy (RMB) is very important in choosing the right treatment for renal tumors for people who have other health conditions or are at higher risk for surgery (47). Figuring out treatment approaches is often possible with the results of a biopsy. The information from RMB can direct important clinical decisions when it is possible to gather direct evidence through tests. RMB also supports treatment planning whenever it is not clear if a partial or complete nephrectomy is needed, as biopsy findings can inform the choice and personalize the plan (48).

RMB is also indicated in atypical imaging features of renal lesions that do not fall into benign or malignant patterns and require tumor sampling for definitive diagnoses. A biopsy is essential in metastatic conditions suspected of being due to RCC. In these cases, histopathological confirmation is established before instituting either targeted therapy or immunotherapy. Although cancer seeding was the original misgiving with RMB, Choy et al. confirmed that it is both safe and feasible with minimal risk (49).

Accurate histopathologic classification of RCC is important in prognosis and development of appropriate therapy. WHO classifies RCC into different groups according to histological and molecular features. Clear cell RCC (ccRCC) is the most frequent subtype, representing 75 to 80% of RCC (48). This subtype is characterized by clear cytoplasm and highly vascular stroma. Clear cell is commonly associated with VHL gene mutation and abnormal angiogenesis. The second subtype is pRCC, representing 10-15% of RCC (50). pRCC appears as either Type 1 with small basophilic cells, with a favorable prognosis, or Type 2 with large eosinophilic cells, with a worse prognosis. Chromophobe RCC (chRCC) represents about 5% of RCC and arises from intercalated cells of the collecting duct (48). It is also characterized by pale cytoplasm with perinuclear halos. Collecting duct carcinoma is rare (1-2%) and highly malignant, arising from epithelial cells, and tends towards an unfavorable prognosis (51). Novel molecular markers have been identified in recent studies with the potential for further stratification of RCC classification and potential targets for therapy. Genetic profiling delineates the potential for individualization of therapy according to the RCC subtype.

Despite the diagnostic utility of RMB, some of the challenges it possesses call for ensuring accuracy and reliability (52, 53). Sampling error is one of the primary issues because renal tumors are highly heterogeneous; hence, one biopsy cannot represent the lesion as a whole (54, 55). Morphological intersections of RCC types make diagnosing even more challenging because some have overlapping morphologies with eosinophilic cytoplasm or hybrid morphology (56). For instance, it is challenging to differentiate chRCC from oncocytoma on small biopsy samples (57, 58). Tumor heterogeneity is another major issue because different regions of the same tumor may have diverse histopathological patterns that may affect diagnostic accuracy (59, 60). Multi-regional sampling is proposed for better accuracy, but it is not practical in everyday clinical practice (55). A combination of histopathology with immunohistochemical stains and molecular analysis has been proposed to overcome such limitations in multidisciplinary approaches (61, 62). Histopathological analysis and the latest technology for sequencing DNA can be improved with artificial intelligence (60). The efficiency of biopsies needs to be raised, and a larger analysis of scarce tissue could enhance the benefits of RMB (63).

### 2.3 Staging and prognosis

RCC treatment and outcomes depend on how advanced the cancer is and the person’s prognosis. RCC is staged using the Tumor, Nodes, and Metastasis (TNM) system, and the size of the tumor, the nearby lymph nodes, and whether metastasis is present are considered (64).

#### 2.3.1 TNM staging system for kidney cancer

RCC is staged and prognosed using the TNM system. The system organizes how the disease is measured by size. Size and local tumor invasion establish T status, while the invasion of regional lymph nodes and metastasis determine N and M status. Classifying cancer is important for deciding on treatment because people with early disease (T1-T2, N0, M0) can often be treated with nephron-sparing surgery. Unlike T1-T2, late diseases (T3-T4 or M1) are treated effectively with systemic therapy (38). Kidney cancers are classified into different risk levels using tumor grade, pathologic subtype, and molecular characteristics.

A greater presence of nucleolar features in a tumor sample is linked to more aggressive growth and a higher grade in the ISUP system. According to the latest research, variations in the VHL, PBRM1, and SETD2 genes are the strongest evidence for tumor growth and how it responds to therapy (48). When RCC is limited to the kidney, more than 95% of patients survive for at least 5 years. Once cancer spreads to other organs, there is a significant decrease in 5-year survival ranging from 0% to 20%. New therapies focused on the immune system, including immune checkpoint inhibitors, have played a big role in better treating metastatic RCC (65). More research is confirming that using molecular profiling improves medical care by making diagnoses more accurate and improving individualized treatment.

#### 2.3.2 Prognostic factors

The prognostic factors of RCC are multidimensional and include anatomical, histologic, clinical, and molecular features. In anatomical classification, the TNM classification has been in use since 1977 to stage diverse solid tumors (48). Histologic features such as RCC subtype, tumor grade, sarcomatoid features, vascular invasion, tumor necrosis, and invasion into perirenal fat influence the prognosis. Incidentally, type I papillary RCC has a lower mortality risk than ccRCC and papillary type II RCC (48). The genes BAP1 and PBRM1 expression on the 3p chromosome are mentioned as being frequently deleted in ccRCC and as an independent predictor of tumor recurrence (65). Patients with BAP1 gene mutations have worse outcomes than those with PBRM1. Even though various reports of molecular markers in the context of prognosis for CAIX, PTEN, CXCR4, methylation status, and expression profile of the genes are present, none have been formally added as part of established prognosis devices (48). Further refines prognosis models with risk stratification needed individually for optimal treatment and outcome.

#### 2.3.3 Survival rates and recurrence patterns

Survival for RCC is highly stage-dependent at presentation, with favorable outcomes for early-stage tumors. The five-year survival rate for RCC is 75% in general but increases to 93% if the disease is caught early and only affects the kidney (37). The presence of renal tumors in nearby lymph nodes or major blood vessels is linked to poorer results, and the prognosis is worse for stage III and IV patients (66). Mattila et al. noted that among patients with ccRCC, in 78.1% of the cohort, five-year survival was 61.9% in stage III (without lymph node involvement), 22.7% in stage III (with positive lymph nodes), and 15.6% in stage IV (48). A greater chance of recurrence is linked to large, high-grade tumors and those with sarcomatoid changes, lymphovascular involvement, or infiltration of perirenal fat. The lungs, bones, liver, and brain are the most common places for cancer metastases. Tumor DNA found in the blood and certain molecular indicators are becoming useful for recurrence monitoring.

### 2.4 Molecular hallmarks of RCC subtypes

The major RCC subtypes are defined by distinct molecular drivers that dictate their pathogenesis, metabolic reprogramming, and immune microenvironments (67, 68). ccRCC is fundamentally characterized by 3p loss leading to biallelic VHL inactivation, resulting in constitutive HIF-1/2α stabilization. This is complemented by frequent mutations in chromatin-remodeling genes (PBRM1, BAP1, SETD2), which significantly influence clinical behavior and therapy response (69). Late progression events include 9p and 14q loss, with advanced disease correlating with increasing somatic copy-number alteration (SCNA) burden and immune evasion mechanisms such as HLA loss of heterozygosity (70). The constitutive HIF activity drives a metabolic shift towards glycolysis and the pentose phosphate pathway while depressing the TCA cycle and β-oxidation; this is often exacerbated by FBP1 loss, collectively shaping therapy response.

Papillary RCC is molecularly heterogeneous. Legacy type 1 tumors are commonly MET-driven, while FH-deficient RCC exhibits a CpG island methylator phenotype (CIMP) secondary to fumarate accumulation, leading to pseudohypoxia and inhibition of α-KG-dependent dioxygenases (71). chRCC classically shows multiple chromosome losses, whereas a metabolically divergent subset (MD-chRCC) lacks these typical losses, displays sarcomatoid features, suppressed Krebs cycle/electron transport chain/AMPK activity, and is associated with poor survival. Other important entities include MiT-family translocation RCCs, which harbor TFE3, TFEB, or MITF fusions; SDH-deficient RCC, which accumulates succinate causing epigenetic dysregulation and reductive carboxylation; and TSC1/2/PTEN/mTOR-altered variants that converge on dysregulated growth-factor/mTOR signaling.

The immune contexture differs markedly by subtype: ccRCC is generally more inflamed yet can be variably Th2-skewed, influencing responses to immunotherapy. In contrast, pRCC and chRCC tend to exhibit lower immune cell infiltration and signatures, with FH-deficient cases representing an exception with an immune profile closer to ccRCC (72). These defining molecular features provide a strong rationale for subtype-tailored targeted therapy (e.g., HIF-2α, MET, and mTOR inhibitors) and critically inform the design of microfluidic liquid-biopsy platforms. Such platforms aim to capture EMT-shifted CTCs, quantify oncometabolite-linked extracellular vesicle (EV) and ctDNA signals, and profile immune markers relevant to immune checkpoint inhibitor (ICI) response.

## 3 Current treatment modalities for kidney cancer

Recent technological progress is making a big difference in kidney cancer treatment by helping to control the disease and preserve kidney function. The techniques adopted in recent years create a situation characterized by oncological efficacy that balances renal function and quality of life. Current practices pay closer attention to personalized care by comparing partial and radical nephrectomy, considering targeted and immunotherapies, and relying more on less invasive surgery.

### 3.1 Surgical interventions

#### 3.1.1 Radical versus partial nephrectomy

Radical and partial nephrectomy are some key surgical treatments used for RCC. The selection of an appropriate method of choice depends on patient factors and the size and location of the tumor. Radical nephrectomy (RN) involves the removal of the whole kidney and is reserved for large, central, or invading tumors. The method is good from an oncologic point of view but is associated with chronic kidney disease (CKD) as well as cardiovascular problems (73). On the other hand, partial nephrectomy (PN) or nephron-sparing surgery is reserved for small renal masses or when preservation of kidney function is of the highest concern. PN is reported to have cancer-specific survival comparable with RN, with minimal risk of long-term renal impairment (74).

Partial nephrectomy (PN) has illustrated better performance on larger tumors (4–7 cm), which is technically feasible because it preserves renal function and decreases dialysis risk (75). PN is, however, risky and is associated with a higher risk of complications such as urine leak and hemorrhage, and it requires expertise in complex surgery. RN is still regarded as the best method when dealing with tumors that invade the renal vein, appear in multiple locations, or have unsuitable anatomy. Studies showing outcomes over time found that, compared to RN, patients with PN have lower rates of CKD and improved chances of surviving, which stresses the need for personalized decisions based on patient and tumor features (76).

#### 3.1.2 Minimally invasive techniques (e.g., laparoscopic and robotic surgery)

Patients with RCC now have improved outcomes because laparoscopic and robot-assisted surgery decreases the risk of major complications after surgery. In more difficult cases, the precision needed for suturing certain tumors can be limited with this technology (77).

Robot-assisted partial nephrectomy (RAPN) allows accurate tumor resection and reconstruction of the kidney with minimal ischemia time and improved preservation of renal function (78). The technique reduces the risk of complications and has less recovery time than laparoscopic PN. This approach makes it the preferred method for complex renal tumors. Although minimally invasive techniques have become standard in localized RCC, their utilization is less frequently seen in large or complex tumors with larger resections. Technology such as image-guided navigation and augmented reality-assisted surgery is crucial in the attempt to maximize accuracy and outcomes with minimally invasive intervention, as illustrated in Lin et al. (79). The choice of approach must balance oncologic safety and functional outcomes.

#### 3.1.3 Complications of surgery and quality of life

Patient factors and the type of procedure influence postoperative nephrectomy complications. Bleeding, infection, urinary fistula, and renal function impairment occur as the major postoperative complications that warrant key consideration. Partial nephrectomy poses an increased risk of delayed hemorrhage and urine leakage compared to radical nephrectomy but with improved long-term preservation of renal function (73). Catastrophic hemorrhage caused by laceration of the renal artery is rare, and postoperative recovery and renal function preservation affect the quality of life (QoL). PN recipients have improved renal function and decreased chronic kidney disease compared to RN recipients. This normalcy appears with a reduced risk of cardiovascular morbidity and dependence on dialysis. Minimally invasive methods improve quality of life by reducing surgery-induced trauma and earlier recovery time (80). Continuous follow-up with imaging, CKD management, and telemedicine enhances long-term recovery and monitoring (81).

### 3.2 Targeted therapies

Targeted therapies have revolutionized metastatic and advanced RCC, with ccRCC being the most common histologic subset. Tyrosine kinase and mTOR inhibitors are the mainstream targeted therapies against cancer cell proliferation and angiogenesis via vascular endothelial growth factor. However, with efficacy in therapy, therapeutic limitations are an inevitable limiting parameter, and therefore, efforts are in progress to overcome therapeutic limitations. This section critically summarizes the literature on the mechanism of action, efficacy, toxicity, and resistance to targeted therapy in RCC.

#### 3.2.1 Tyrosine kinase inhibitors and mTOR inhibitors

The mTOR inhibitors and TKIs are pillars of targeted RCC therapy, which act by interrupting crucial pathways that are responsible for the development of cancer. Two of the targets for TKIs are the VEGF receptor (VEGFR) and the platelet-derived growth factor receptor (PDGFR). Tumor vascularization is inhibited effectively in their application by sunitinib, axitinib, and pazopanib through receptor inhibition. Subsequently, nutrient and oxygen starvation leads to the inhibition of cancer growth (82). The inhibition develops newer TKIs that selectively target MET and AXL signal transduction pathways, which mediate VEGFR inhibition-induced growth and metastasis of cancer (80).

MTOR inhibitors everolimus and temsirolimus target the PI3K/AKT/mTOR pathway vital to cell growth, division, and metabolism (83). In RCC, mutations in the VHL gene lead to aberrant activation of this pathway, causing uncontrolled tumor growth due to transmembrane receptor tyrosine kinases (84). The superior response rates and higher survival rates of TKIs and immune checkpoint inhibitors make mTOR inhibitors reserved as a second-line treatment (82). Combination regimens are being studied to enhance outcomes and overcome resistance.

#### 3.2.2 Clinical efficacy and side effects of targeted therapies

Large randomized trials have established the efficacy of TKIs in metastatic RCC. In the COMPARZ trial, pazopanib demonstrated non-inferior efficacy to sunitinib, with comparable median progression-free survival (PFS 8.4 vs 9.5 months) and overall survival (OS 28.2 vs 29.3 months), but a more favorable tolerability profile and better patient-reported quality of life (85). Clinically, this indicates that both agents achieve similar disease control, and the choice between them is primarily guided by side-effect profiles, comorbidities, and patient preference rather than small numerical differences in median survival.

The METEOR study confirmed cabozantinib as an effective second-line option after VEGF-targeted therapy, showing significant improvements in PFS (7.4 vs 3.9 months) and OS (21.4 vs 16.5 months) compared with everolimus (86). Although the absolute survival gain was modest, it translated into clinically meaningful benefit for previously treated patients, supporting cabozantinib as the preferred second-line standard while emphasizing the importance of balancing efficacy with toxicity and patient quality of life.

TKIs commonly cause hypertension, diarrhea, fatigue, and hand-foot syndrome, which often necessitate dose adjustment or discontinuation. Comparative analyses demonstrate that pazopanib is better tolerated than sunitinib, particularly with respect to fatigue and gastrointestinal toxicity (85, 86). In contrast, mTOR inhibitors such as everolimus and temsirolimus are associated with adverse events including stomatitis, hyperglycemia, dyslipidemia, and pneumonitis, which complicate prolonged use (87). Given that efficacy differences among these targeted agents are small in the first-line setting and moderate in later lines, individualized therapy based on toxicity profile, comorbidity, and patient-reported outcomes remains the cornerstone of clinical decision-making (85–87).

#### 3.2.3 Resistance mechanisms and strategies to overcome them

The greatest challenge in treating RCC is the development of acquired resistance to targeted therapy in most victims. Changes in VEGF signaling are largely responsible for TKI resistance through the activation of other pathways for neo-angiogenesis and adaptation of the tumor cells to hypoxic conditions. MET and AXL overexpression has been reported as the primary mechanism of tumor resistance to inhibition of VEGF through the introduction of multi-kinase drugs such as cabozantinib and the development of lenvatinib targeting both the VEGF and MET/AXL pathway in order to delay the onset of resistance (88). In addition, hypoxia-induced factor-1α overexpression is also involved in TKI resistance through VEGF-independent angiogenesis, which further complicates the problem.

Resistance to mTOR inhibitor therapy is mainly a result of activation of the PI3K/AKT pathway in compensation, which preserves tumor growth and survival in the presence of mTOR inhibition. Preclinical efficacy has been demonstrated with combined approaches to targeting both upstream PI3K and mTOR, but translation to the clinic has been hampered due to problems with toxicity (89). Combination therapy has been an important method in overcoming resistance to therapy. The KEYNOTE-426 trial showed that axitinib plus pembrolizumab increased overall survival (OS) over that with sunitinib alone (90). Triple combinations of nivolumab and cabozantinib with ipilimumab are under investigation to overcome resistance.

### 3.3 Immunotherapy

#### 3.3.1 Immune checkpoint inhibitors

Immune checkpoint inhibitors have now made it possible to effectively manage metastatic RCC. The function of these inhibitors is to stop the programmed cell death protein-1 and PD-L1 from working so that the immune system handles tumor cells better. When PD-1 and PD-L1 interact, it usually means the T-cell system slows down, making it harder for the immune system to notice cancer cells. Stopping this pathway with checkpoint inhibitors revives T-cells and raises the ability to fight cancer. Patients diagnosed with advanced kidney cancer who have PD-1 or PD-L1 expression often show improved survival results after being treated with nivolumab (anti-PD-1) or atezolizumab (anti-PD-L1) (91). These results allow doctors to improve cancer treatment and show that biomarkers can help them decide the right treatment plan for each patient.

RCC patients can receive nivolumab as an important immune checkpoint inhibitor. An analysis from the CheckMate 025 trial found that OS was better among patients taking nivolumab than among those taking everolimus in treatment after failing initial therapy (86). Pembrolizumab and TKIs were evaluated in KEYNOTE-426, with the study revealing these combinations substantially improved OS and PFS compared to sunitinib used alone (92). Favorable results from using atezolizumab together with targeted therapies that target the VEGF pathway were observed in some RCC patient groups. Even so, it seems that some patients do not respond positively to ICIs. Development is still needed for predictive biomarkers and the combined use of therapies to achieve better results.

#### 3.3.2 Combination therapies and their outcomes

Adding ICI therapy to TKIs or mTOR inhibitors or using both at the same time increases the efficiency of treatment. When these approaches are used together, they prevent tumor growth and, at the same time, reduce the chance of resistance to treatment. The use of nivolumab and ipilimumab during the CheckMate 214 trial resulted in a lasting response and better survival than sunitinib for patients with intermediate - or poor-risk RCC, with the effect being more serious in patients whose tumors expressed PD-L1 (93).

Using both ICIs and TKIs together in treating RCC has become very useful. Researchers discovered in KEYNOTE-426 that pembrolizumab (anti–PD–1) combined with axitinib resulted in better overall survival (OS), progression-free survival (PFS), and objective response rate (ORR) for people with advanced RCC who have never received treatment for their disease (90). It was also shown in JAVELIN Renal 101 that avelumab worked better with axitinib since, together, they achieved higher responses and maintained disease control longer within the 95% confidence interval (94). They make treatment more effective and help control immune-related health problems, such as colitis, pneumonitis, and changes in the endocrine system. Additionally, trials are testing new therapies by combining ICI, TKI, and mTOR inhibitor drugs with the usual treatment strategy.

#### 3.3.3 Biomarkers for predicting immunotherapy response

Predicting the patient’s reaction to immunotherapy is a significant problem when treating RCC. It reveals that many different scenarios can influence the immune system and the course of tumors in patients. Difficulty in finding biomarkers to predict if kidney cancer patients have a good response to immunotherapy limits its use. Although PD-L1 plays a role in other diseases, it gives uncertain results in kidney cancer, according to Reimold et al. (95). While presenting the observable association of increased response to ICI through tumor evasion (96), there are also instances without any statistically significant association.

Tumor mutational burden (TMB) has not shown much promise as a predictor for how patients with RCC might respond to immunotherapy. The usual low TMB in RCC lowers its value as a good biomarker for immunotherapy, unlike melanoma and similar forms of cancer (97). Research has been carried out on gene expression signatures such as angiogenesis and inflammatory genes to stratify who would benefit most from ICIs over TKIs based on their genomics and transcriptomics (98). Findings validate that microenvironment features, such as the availability of TILs, myeloid-derived suppressor cells, and Tregs, influence the prediction of responses to immunotherapy by patients (99). Circulating biomarkers, such as cytokines in the circulation, exosomal PD-L1, and profiling of circulating tumor DNA (ctDNA), are superior and accurate non-invasive markers used to predict responses and resistance to therapeutic intervention (100). Advances in single-cell RNA sequencing and spatial transcriptomics promote precise biomarker-guided therapy selection, ensuring maximum efficacy of immunotherapy in RCC.

### 3.4 Emerging therapies and clinical trials

#### 3.4.1 Novel therapeutic targets (e.g., HIF-2α inhibitors)

HIF-2α inhibitors are one of the most promising new treatments for RCC. As ccRCC is frequently associated with von Hippel-Lindau (VHL) gene loss-of-function mutations with subsequent HIF-2α accumulation, pathway-targeted inhibition has emerged as a new therapeutic avenue. Belzutifan, the first-in-class HIF-2α inhibitor, has demonstrated promising activity in patients with VHL-related RCC and sporadic ccRCC. The phase II LITESPARK-004 trial proved that belzutifan had an objective response rate (ORR) of 49% in RCC with VHL disease and a good safety profile (101).

Research is identifying new targets for inhibition as a key contribution to the available literature. MET and AXL inhibitors such as cabozantinib are crucial in overcoming the resistance to therapies targeting VEGF and immune checkpoint therapies (102). The use of epigenetic modulators such as bromodomain and extraterminal (BET) proteins to induce oncogenesis is being researched. BET proteins influence gene expression and the likelihood of developing kidney cancer, and their utilization as inhibitors is a novel approach to anticancer intervention (103). Additional immunomodulators, such as bispecific antibodies and IL-2 pathway agonists, are used in the process of developing next-generation regimens for immunotherapy. The therapies elicit the best immune response with minimal side effects, opening the window for resistant RCC patients.

#### 3.4.2 Gene therapy and advances in personalized medicine

Gene and individualized therapy are novel RCC treatments that use genetic profiling to individualize treatments to the patient’s requirements. Next-generation sequencing and transcriptomics have revealed RCC molecular subtypes with distinct therapeutic weaknesses. For instance, PBRM1 mutations are used as predictive biomarkers in understanding the response to immune checkpoint therapies (104). The identification of BAP1 mutations is associated with higher progression-free survival benefits after patients receive ICI immunotherapies.

Gene therapies are used to target TME and enhance the immune response. CRISPR genome editing is used to edit the tumor suppressor genes and immunogenic cancer. Recent studies in RCC models have shown that CRISPR-mediated knockout of immunosuppressive pathways, such as PD-L1, can significantly enhance T-cell cytotoxicity and promote stronger antitumor immune responses by increasing tumor cell visibility to the adaptive immune system (105). The CRISPR genome editing technique increases the immunogenicity of cancer cells while promoting treatment outcomes. Additionally, CRISPR has been explored to target metabolic and hypoxia-related pathways in RCC, including components of the VHL/HIF axis, which contributes to an immunosuppressive tumor microenvironment. By disrupting these pathways, CRISPR can alter cytokine signaling and enhance immune infiltration, further improving responsiveness to immunotherapies (106). This reduces the immunosuppressive effects and eliminates metastatic tumors (107). Using genetically modified oncolytic viruses helps destroy RCC cells and encourages the body’s immune system to fight cancer. The function of viruses is to replicate often in cancer cells, resulting in the rupture of those cells and the release of substances that trigger the immune response. Real-time changes in cancer treatment are guided by using liquid biopsies to monitor therapy. Experts consider both exosomal RNA and ctDNA at early stages to guide treatment and find out how the cancer might resist those treatments. These therapies make it possible to apply customized and flexible treatment to RCC (108).

#### 3.4.3 Overview of ongoing clinical trials and their potential impact

Researchers are testing recent medications in clinical trials to help treat renal cell cancer better and more effectively. The LITESPARK-011 trial program is an important case where using belzutifan together with targeted therapies was studied in patients facing advanced RCC. Results suggest that targeting HIF-2α could represent a useful therapy before or after other treatments for advanced RCC that were not controlled with PD-L1 therapy (109). In the COSMIC-313 trial, advanced kidney cancer patients with residual disease were included to find out if the combination of cabozantinib, nivolumab, and ipilimumab was safe and effective. A combination drug approach to treating advanced cancer showed patients enjoyed increased progression-free survival, although this approach brought side effects (110).

Among the most important changes is CAR-T therapy, which has helped treat RCC greatly and has shown great improvements for hematologic malignancies. The technique requires the engineering of the genetic T-cells of the patient to recognize and eliminate cancerous tissues from the body. Nonetheless, the mentioned technology has faced difficulties in tailoring the treatment of solid tumors such as RCC due to the immunosuppressive tumor microenvironment. Clinical trials are evaluating genetically engineered T-cells targeting CAIX, an antigen highly overexpressed in ccRCC (111). The findings of trials indicate potential for clinical application and will contribute immensely to available evidence, as noted by Khan et al. (112). Tumor neoantigen-targeted vaccines are being examined as a unique treatment approach to boost the immune system and lead to enduring anti-tumor responses that could give patients more control over their disease (113). These results will largely shift future treatment for RCC, moving away from generic therapy towards treatments designed for specific features. Therefore, doctors can give patients tailored treatments based on their molecular and immune system features when new medicines and treatment steps are developed.

## 4 Biomolecular techniques in kidney cancer detection

### 4.1 Molecular pathology and biomarkers in kidney cancer

#### 4.1.1 Genomic alterations in kidney cancer

Researchers closely examine how changes in the Von Hippel-Lindau (VHL) function of the ccRCC tumor suppressor gene affect medical conditions. In the absence of VHL gene protein, the levels of both HIF-1α and HIF-2α increase in the HIF pathway. Additionally, as tissues degenerate, blood vessels increase, more cells are produced, and the way cells metabolize can change. The vast majority of ccRCC patients have VHL gene changes and epigenetic changes, which cause their tumors to become difficult to treat (114). Its unique role in renal cancer makes VHL a key point for both detecting and treating the disease. New drugs inhibiting HIF-2α, for example, belzutifan, are interesting therapies that further support available evidence for treatment.

Significant genetic alterations such as PBRM1, SETD2, and BAP1 are common in ccRCC. PBRM1 is mutated in about 33% of ccRCC and plays a role in chromatin remodeling, which are missense mutations (115). PBRM1 is linked with enhanced responses to immune checkpoint agents. BAP1 mutations are attributed to enhanced aggressiveness of the tumor and poor survival. Angori et al., in the genomic evaluation of papillary RCC, revealed that mutations of MET are an important tumorigenesis driver. MET encodes for the hepatocyte growth factor receptor and is mutated in type 1 pRCC, giving rise to constitutive activation of pro-survival signals (116). MET antagonists, such as cabozantinib and savolitinib, influence MET-driven tumors and expression. Type 2 pRCC is genomically heterogeneous, with alterations seen with CDKN2A, NRF2, and FH, typical of hereditary leiomyomata and RCC.

#### 4.1.2 Proteomic and metabolomic biomarkers

The pathogenesis of kidney cancer is impacted by proteomics, leading to differentially expressed proteins being recognized as markers for prognostication and diagnosis. CAIX is a major protein studied in ccRCC, which is overexpressed because of loss of the von Hippel-Lindau (VHL) gene. CAIX is used to indicate tumor metabolism under hypoxia and highlight association with high-risk features. Thus, CAIX is considered an informative marker of patient selection for immunotherapy and targeted therapy treatments (117).

In kidney cancer, there is an overexpression of Kidney Injury Molecule-1, which is linked with the progression of the tumor (118). KIM-1 serves as a valuable element in the early diagnosis of RCC using urine-based assays, a minimally invasive diagnostic method. TIMP-1, a Tissue Inhibitor of Metalloproteinases-1, is used as a marker of poor survival and high metastatic capacity. Metabolic profiling is utilized in distinct metabolic reprogramming in RCC. The aberrant metabolism of glucose and lipids enables the consideration of putative biomarkers such as metabolites like lactate, fumarate, and succinate. In the case of fumarate accumulation, the biomarker is used to indicate FH-deficient RCC and, in turn, to identify metabolic signatures for hereditary kidney tumors (119).

#### 4.1.3 Role of biomarkers in prognosis and treatment selection

Biomarkers improve prognosis and guide therapeutic decisions in RCC. Traditionally, risk stratification was based on clinical factors such as tumor size, stage, and histologic grade. However, the incorporation of molecular biomarkers significantly improves prognostic accuracy. The IMDC risk model, which places patients into poor-risk, intermediate-risk, or favorable-risk categories, employs molecular markers such as LDH (lactate dehydrogenase) and NLR (neutrophil-to-lymphocyte ratio) to predict survival outcomes (120). High LDH levels reflect glycolytic activity and tumor hypoxia and are associated with poor prognosis in kidney cancer.

The discovery of immune checkpoint inhibitors made patient selection based on biomarkers a requirement for immunotherapy. PD-L1 expression is an unreliable predictor for ICI response in RCC (121). However, newer biomarkers, including T-cell-inflamed gene expression signatures, TMB, and interferon-γ pathways, are better predictors of response to immunotherapy.

### 4.2 Liquid biopsy and circulating biomarkers

#### 4.2.1 Advantages of liquid compared to traditional biopsy

**Figure 4:**
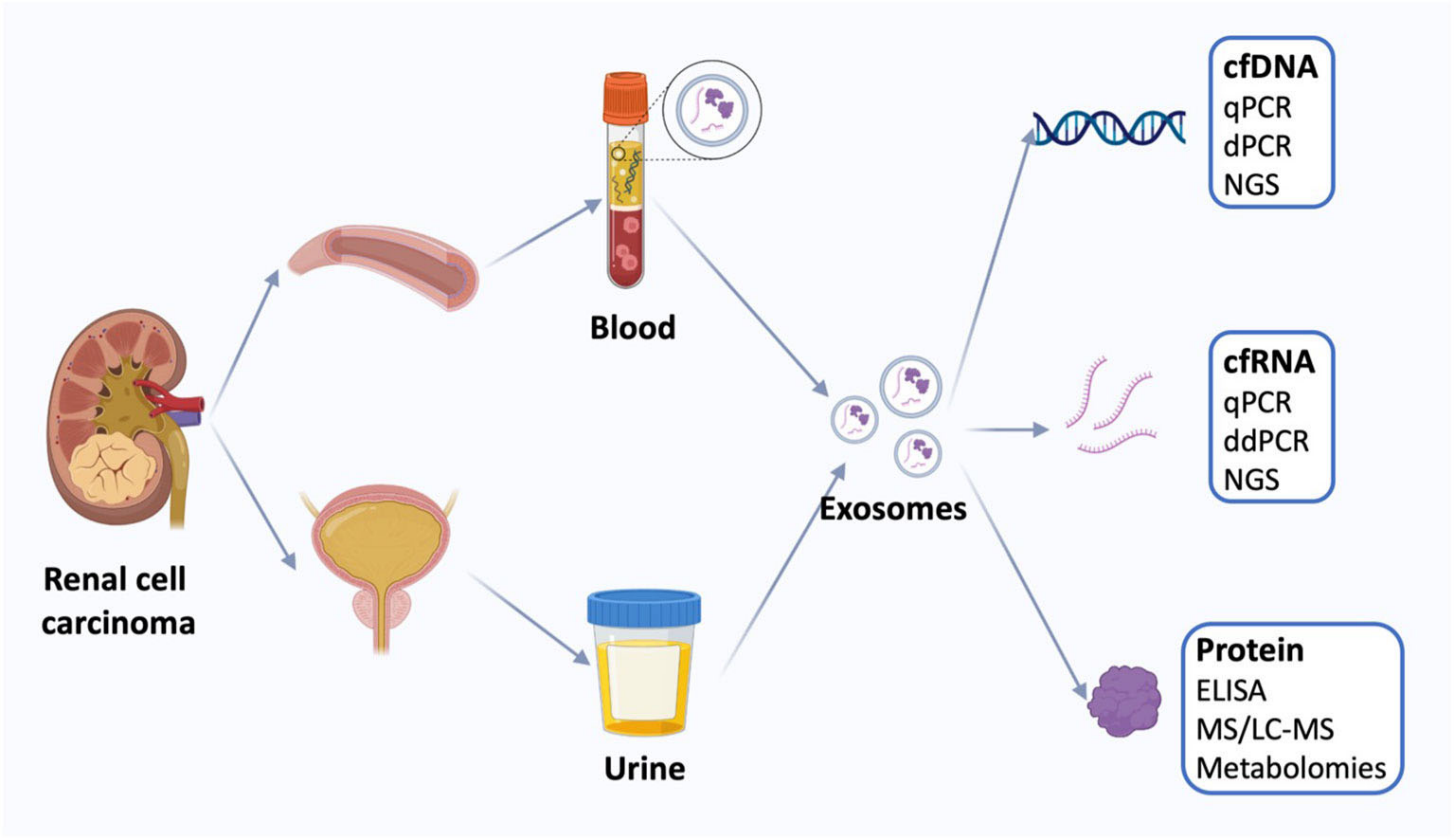
Liquid biopsy of renal cell carcinoma. Liquid biopsies for kidney cancer use mainly blood and urine samples, including exosomes and the cell-free DNA (cfDNA), cell-free RNA (cfRNA), and proteins. Quantification of circulating tumor markers at a single time point may be useful for disease staging and prognosis using a variety of techniques. cfDNA, cell-free DNA; qPCR, quantitative polymerase chain reaction; dPCR, digital polymerase chain reaction, NGS, next-generation sequencing; cfRNA, cell-free RNA; ddPCR, droplet digital polymerase chain reaction; ELISA, enzyme-linked immunosorbent assay; MS, Mass spectrometry; LS-MS, liquid chromatography-MS. (Reproduced from (30), under CC BY 4.0 license).

Liquid biopsy based on non-invasive methods in the treatment of kidney cancer is preferred compared to conventional tissue biopsy (122). Liquid biopsy measures tumor-derived materials in body fluids, including plasma, urine, and blood, instead of using operation- or needle-biopsy-harvested tissue (**Figure 4**). The biopsy utilizes tumor-derived elements, including ctDNA, cells, exosomes, and microRNAs, which carry tumor gene status and drug responsiveness information (123). Liquid biopsy, as a new research field, is geared towards enhanced sensitivity, specificity, and clinical relevance. Conventional tissue biopsy is affected by the invasiveness of the procedure, sampling bias, and failure to reflect tumor heterogeneity and dynamic tumor biomarker changes.

Liquid biopsies eliminate the need for surgical or needle biopsies. Minimizing the patient morbidity and complications of the surgical procedures. The biopsy is useful for patients with metastatic or inoperable renal carcinoma, for which repeated biopsies of multiple organs are not feasible. The easy withdrawal of blood or urine samples at regular intervals makes the repeated assessment of tumor evolution and response to treatment feasible. Traditional biopsy only samples a portion of a tumor and is susceptible to sampling bias (122). Liquid biopsy is used to acquire genetic content from various sites within a tumor and attain a representative molecular image of the disease.

#### 4.2.2 Detecting circulating tumor DNA and exosomes

The detection and analysis of ctDNA in kidney cancer patients has tremendous potential for early detection, prognosis, and therapy monitoring. ctDNAs are circulating tumor-derived DNA fragments that are shed into the circulation due to apoptosis and necrosis of cancer cells. Next-generation sequencing (NGS) and polymerase chain reaction (PCR)-based methods have improved the sensitivity and specificity of ctDNA detection. ctDNA carries mutations common in RCC, as Parums showed (124). Quantitation of ctDNA levels is correlated with tumor burden and metastatic spread and is a valuable biomarker for disease monitoring.

Exosomes are extracellular nanovesicles that originate from tumor cells with DNA, RNA, protein, and lipid components. Exosomes are involved in intercellular communications and in controlling tumor growth and immune evasion. Exosomal RNA and protein contents represent potential biomarkers used to detect cancer occurrence and predict responses to treatments. mRNA in individual exosomes differs in expression among different RCC patients, providing clinical and prognostic value (30). Exosomes have the potential as a delivery system for therapies to transfer immunomodulatory drugs into cancer cells directly.

#### 4.2.3 Challenges in standardizing liquid biopsy techniques

Despite liquid biopsy being widely embraced in RCC, there are existing challenges that impede the clinical application of the technique. The main limitation entails the low levels of ctDNA in RCC patients, particularly in the early stages of cancer. Because RCC secretes relatively low levels of ctDNA compared to highly proliferating tumor models, detection becomes a challenge. Highly sensitive analytical instruments and tumor-guided analysis of plasma are crucial in enhancing the detection rates of ctDNA (125). The analysis achieves high technical sensitivity for identifiable tumors with potential downsides when relatively new tumors are involved. Analytical and pre-analytical variations impede reproducibility as well as the reliability of liquid biopsy analyses in clinical implementation. The variation due to extraction, sequencing technologies, and bioinformatics pipelines challenges the process of data interpretation (126). This proves the importance of standardizing liquid biopsy methods in favor of proper, comparable, and accurate results in clinical practice.

Liquid biopsy assays have faced ongoing regulatory approvals and clinical trials. Although liquid biopsy has been extremely promising in research, translation into routine clinical practice is only possible with large-scale clinical trials that will ensure full-scale validation. The FDA and EMA require robust evidence of the clinical usefulness and economic value of liquid biopsy tests before routine use is approved. In addition, integration into clinical workflows currently being applied is logistically and economically challenging since healthcare delivery systems must change if novel diagnostic technologies are to be adopted. Ongoing and future studies of liquid biopsy for use with kidney cancer are aimed at enhancing assay sensitivity, discovering new circulating biomarkers, and integrating multi-omics platforms (127). Combining liquid biopsy with additional omics technologies, including transcriptomics and proteomics, is anticipated to increase diagnostic accuracy and further illuminate tumor biology. As liquid biopsy technologies continue to advance, application with kidney cancer is expected to expand with non-invasive, real-time disease detection, monitoring, and personalized treatment selection.

### 4.3 Emerging biomolecular technologies

#### 4.3.1 Single-cell sequencing and spatial transcriptomics

Single-cell sequencing (SCS) has revolutionized the study of tumor heterogeneity by enabling genetic and transcriptomic analysis at single-cell resolution. Unlike bulk sequencing, which averages the signal across a mixture of different cell types, SCS allows precise exploration of a tumor’s diverse cellular landscape. This approach has revealed distinct genetic alterations, variable drug responses, and rare subpopulations of malignant cells that harbor unique mutations, resistance pathways, or metastatic potential. In RCC, single-cell RNA sequencing (scRNA-seq) has been instrumental in defining the gene expression profiles that characterize aggressive tumor phenotypes (128). Recent studies have applied scRNA-seq to multiple pathological subtypes of RCC, including ccRCC, pRCC, and chRCC variants (129). By profiling thousands of tumor cells, these analyses identified distinct subclusters expressing novel tumor-specific markers such as SPOCK1, PTGIS, REG1A, CP, and SPAG4, along with specialized endothelial and fibroblast populations that contribute to tumor progression and microenvironmental remodeling. Similarly, a comprehensive single-cell atlas of renal cancers was developed (130), highlighting the heterogeneous composition of malignant, immune, and stromal cells, and linking immune infiltration patterns with therapy response and prognosis through cytokine- and chemokine-mediated signaling. Reconstruction of tumor evolutionary trajectories through SCS has also clarified the clonal evolution and mechanisms of drug resistance in kidney cancer. For instance, a recent large-scale study revealed pronounced metabolic heterogeneity in ccRCC using single-cell transcriptomics across nearly one million cells, identifying glycolytic and lipid-metabolic reprogramming clusters associated with differential drug sensitivity (131). The characterization of distinct tumor cell populations and their responsiveness to targeted agents or immunotherapies provides a foundation for tailoring treatments to individual cases of advanced or refractory RCC. Furthermore, single-cell profiling of metastatic RCC has identified metastasis-associated transcriptional signatures and immune exhaustion markers, offering new insight into the molecular basis of tumor dissemination and therapeutic resistance (132).

**Figure 5:**
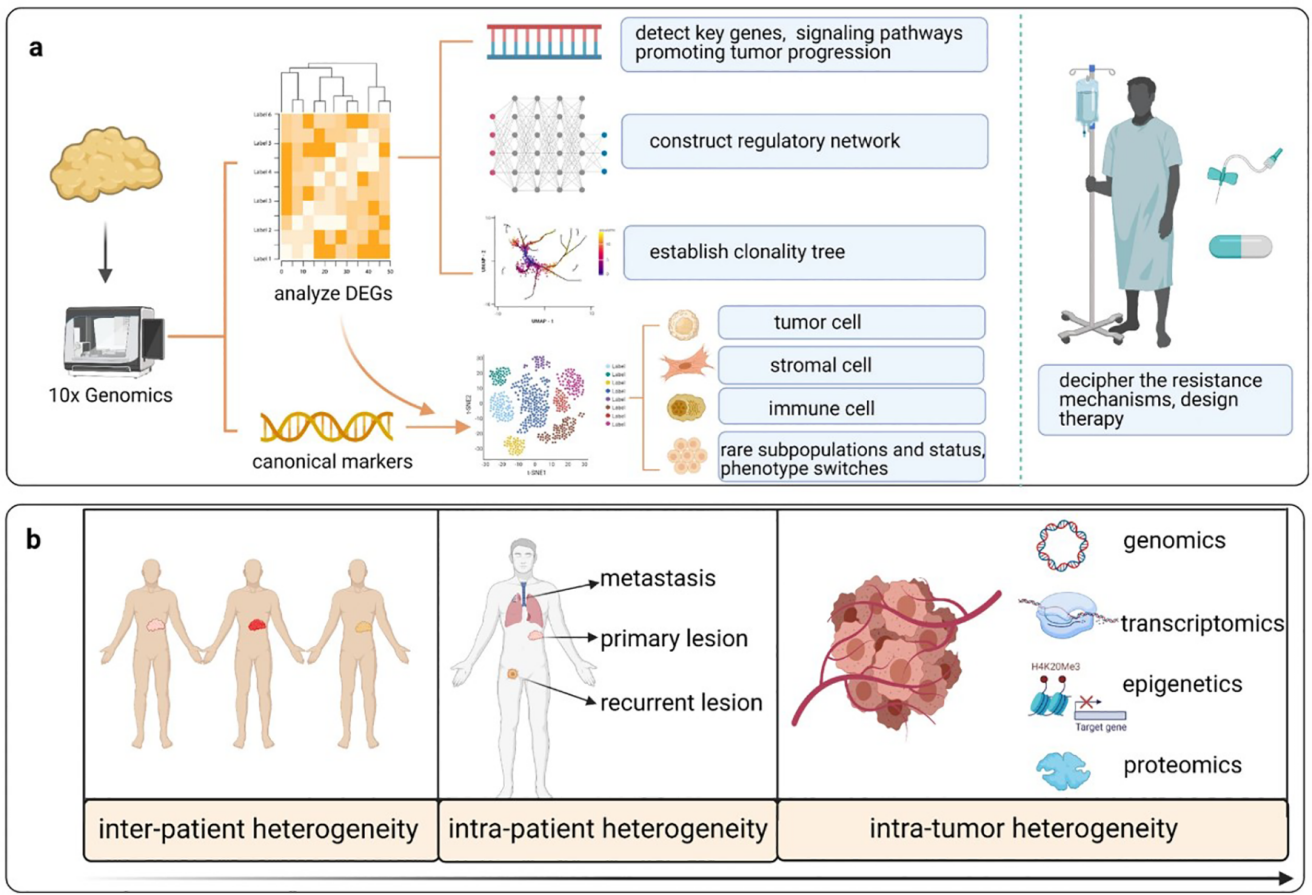
Application of single-cell sequencing in delineating tumor heterogeneity and designing novel targeted therapies for patients with various tumors. **(a)** Single-cell sequencing can be used to analyze differentially expressed genes (DEGs), thereby detecting key genes and signaling pathways that are altered during tumor progression and constructing a regulatory network and clonality trees within tumor lesions. When DEGs are combined with canonical markers, the cells are clustered, which enables the identification of rare subpopulations, cell states and phenotype switches during tumor progression. Interrogation of the tumor microenvironment (TME) and heterogeneity enables the disclosure of therapeutic resistance mechanisms and the design of novel therapies. **(b)** Single-cell sequencing explores tumor heterogeneity at distinct levels, including the population, individual cell, tissue and molecular levels. (Reproduced from (133), under CC BY 4.0 license).

Characterization of distinct tumor cell populations and sensitivity or responsiveness to targeted agents or immune therapies enables clinicians to tailor treatments to individual cases of advanced or refractory RCC. Spatial transcriptomics has similarities with single-cell sequencing. The similarities are that it maintains spatial gene expression architecture within tumor microenvironments. In contrast with scRNA-seq, which requires the dissociation of cells before sequencing, ST does not lose spatial resolution. This makes it feasible to map tumor histological sections with gene expression profiles. Notably, it is important because the architecture of a tumor and interaction with surrounding stroma and immune infiltrating cells dictate disease outcome and treatment response. The innovation of ST platforms, including Slide-seq, makes it possible to have a high-resolution mapping of molecular features of RCC tumors (128). Combining spatial transcriptomics with single-cell sequencing will provide an integrated perspective on kidney cancer biology and the identification of novel targets for therapy as shown in **Figure 5**.

#### 4.3.2 Advances in next-generation sequencing

Next-generation sequencing is a useful tool in the research of kidney cancer through global genomic, transcriptomic, and epigenomic tumor profiling. NGS technologies such as whole-exome sequencing, whole-genome sequencing, and RNA-seq enable the identification of the genetic alterations in RCC. The mutations drive tumorigenesis and are useful biomarkers for prognosis and treatment selection. A significant advance in NGS is the creation of targeted sequencing panels for kidney cancer that enable concurrent and cost-effective detection of clinically actionable mutations, fusion genes, and copy number variations (134). Targeted NGS assays have been utilized to identify MET mutations in papillary RCC. NGS-based techniques have been used to explore epigenetic changes, such as DNA methylation changes, to uncover details on novel epigenetic biomarkers for the early detection of RCC.

RNA-seq transcriptomic analysis pinpoints gene expression patterns concerning RCC subtypes, malignancy, and immune imbalances. The sequencing identifies overexpression of checkpoint molecules such as PD-L1 and CTLA-4 in relation to the specific percentage of RCC tumors that form a basis for immune checkpoint inhibitor therapy. NGS in liquid biopsy detects ctDNA and exosomal RNA in plasma from RCC subjects. The minimally invasive procedure provides real-time monitoring of tumor growth, drug responses, and resistance early in the course of treatment. Third-generation sequencing platforms, such as nanopore sequencing and single-molecule real-time (SMRT) sequencing, increase efficiency and accuracy in RCC genomics. The platforms allow for long-read sequencing, in which complex structural variations can be established. The system has alternative splicing events that cannot be properly determined from short-read sequencing (135). The cost of sequencing is drastically reducing while the technology of bioinformatics programs is improving. This maximizes NGS as a tool in precision medicine in genomically diverse cancers.

#### 4.3.3 Integration of multi-omics data for comprehensive analysis

Multi-omics integration is a crucial tool used in kidney cancer analysis and diagnosis. The analysis involves combining genomic, transcriptomic, proteomic, metabolomic, and epigenomic data for a global view of tumor biology (136). The capturing of complex molecular interactions in RCC reveals mechanisms of tumor aggressiveness, novel biomarkers, and targets for therapy. In oncology, various molecular information is combined with patient prediction to respond to targeted immunotherapy. Metabolomic profiling has established subtype-restricted reprogramming of metabolism for RCC, which shapes drug resistance and interaction with the tumor environment. Epigenomic and transcriptomic integration creates aberrant patterns of DNA methylation as biomarkers of prognosis (137). Aberrant methylation of genes such as *VHL, RASSF1A*, and *CDH1* can be detected in plasma, enabling early tumor detection and differentiation between malignant and benign renal lesions. Methylation-based ctDNA assays show higher sensitivity than mutation analyses, making them valuable for disease monitoring and recurrence prediction. Integrating methylation analysis with microfluidic platforms may further enhance early detection and personalized surveillance in RCC (125, 138, 139). Computational developments employing artificial intelligence and machine learning make analytical potential possible in multi-omics datasets, allowing predictive models to explore drug responsiveness and RCC progression. The development of computational tools supports the integration of multi-omics, which is now a key feature in developing personalized treatment regimens.

### 4.4 Microfluidic CTC isolation in RCC: unresolved technical obstacles

Unlike breast or lung cancer, where EpCAM-positive, higher-shedding CTCs and abundant ctDNA repeatedly enable robust liquid-biopsy workflows, RCC presents a low-shedding, EMT-skewed phenotype with frequent EpCAM loss and lower circulating analyte abundance. These biology-driven differences create distinct engineering/validation requirements for microfluidic CTC platforms intended for RCC rather than for “generic” oncology devices.

To contextualize feasibility in RCC, recent microfluidic CTC studies and reviews report spiking recoveries typically 60-90% at 10–100 cells·mL^−1^, with clinical LODs in the low single-digits per 7.5–10 mL after enrichment and immunofluorescence confirmation. Residual WBC backgrounds of ~10^2^-10^3^·mL^−1^ are common without leukodepletion or cascade designs, which can limit downstream single-cell work. RCC cohorts additionally show lower baseline CTC yields than breast/lung, pushing platforms toward higher input volumes, parallelization, or gentle recirculation to preserve viability (e.g., 28, 125, 140, 141).

## 5 Microfluidics in kidney cancer research

### 5.1 Principles of microfluidics in cancer detection

#### 5.1.1 Fundamentals of microfluidic technology

Microfluidics is an extremely advanced technology for managing extremely minute amounts of fluids in the research and diagnosing of cancer. Microfluidics is founded on a principle of accuracy based on micron-scale channels, with integrated sensors, electrodes, and functional elements that enable complex biochemical interactions (142).

Microfluidic devices enable cancer detection by purifying and diagnosing small biological samples with greater accuracy and efficiency. Advanced technology offers significant advantages with the use of microfluidic platforms, unlike traditional diagnostic techniques, which have slow analysis rates and large sample sizes. Microfluidic systems use basic physics, including electrophoresis, to perform high-resolution manipulation and separation of cells and tissue constituents (29). The devices also achieve versatility from fabrication using various materials, including glass and thermoplastics, to enable customization for applications, such as research in kidney cancer.

#### 5.1.2 Advantages of microfluidics in cancer research

The application of density gradient centrifugation is impeded by low sensitivity and specificity for capturing CTCs. This challenge is addressed by the addition of microfluidic devices that promote the application of physical attributes of CTCs, increasing efficiency (140). The use of label-free methods such as inertial microfluidics preserves cell integrity for further use of the extracted samples.

Microfluidics examines extracellular vesicles (EVs), namely exosomes and microvesicles, in kidney cancer diagnostics to evaluate the role of exosomes in tumor growth and intercellular interactions. The diagnostic devices separate EVs from blood, urine, and other body fluids, which allows research into their constituents. Single-vesicle EV analysis describes drug resistance and heterogeneity of kidney cancer (143). Microfluidic devices facilitate high-throughput drug screening for targeted testing and treatment planning. Reproducibility is exhibited by loading a sample tumor cell into a microfluidic chip to screen oncologic chemotherapeutic agents and targeting therapeutics in a controlled microenvironment. This requires the use of a replication of the tumor microenvironment from the organ-on-a-chip model with a real-time observation of drug interactions and drug resistance (144). The research findings promote reductions in treatment regimens and simplification of clinical outcomes in kidney cancer.

Microfluidic technology is associated with automation and the miniaturization of complicated lab procedures. The integration of various functions like sample preparation, separation, analysis, and detection into one microfluidic chip enables scientists to simplify analysis and diagnosis processes (27). The integration of capabilities eliminates labor-intensive procedures, reduces errors, and enhances the reproducibility of experimental data. Despite the advantages of microfluidics, challenges are hindering the extensive use of microfluidic technology to study kidney cancer and medical diagnostics. The main challenges are standardization of the fabrication process, the validation of microfluidic assays, and regulation support (145). Overcoming these issues is crucial to accelerate the adoption of microfluidic devices in clinical practice. The microfluidic platform functionality is enhanced by continuous microfabrication innovation, nanotechnology, and bioengineering.

### 5.2 Applications of microfluidics in kidney cancer

Microfluidics enables accurate, effective, and inexpensive tumor biomarker analysis for transforming cancer studies. The devices form a focal point for CTC isolation and identification. Such applications support exosome and EV detection, drug screening, and resistance testing (30). The applications are crucial for early detection, monitoring, and personalization of prognosis regimens. This section provides a comprehensive overview of recent developments in microfluidic platforms in kidney cancer studies.

#### 5.2.1 Isolation and analysis of circulating tumor cells

CTCs are released into circulation from the initial tumor and are the best biomarker for metastasis, prognosis, and drug responsiveness for kidney cancer. Conventional methods of isolating CTC using density gradient centrifugation and immunomagnetic bead separation are limited by sensitivity, purity, and viability. Microfluidic devices are a more sophisticated tool that harnesses biophysical and biochemical features of CTC for high-efficiency capturing and subsequent analysis. Microfluidic isolation methods of CTC could be divided into label-based and label-free categories. Label-based methods are based on EpCAM or other tumor-specific antigens targeting antibody-coated surfaces. Kidney cancer CTC would, however, go through epithelial-to-mesenchymal transition (EMT) and lose EpCAM expression, which interferes with the efficiency of such a method (141). Label-free microfluidic methods involving size-based filtration, inertial microfluidics, and deterministic lateral displacement are an alternative for the selective isolation of CTCs based on size, deformability, and hydrodynamical features. Inertial microfluidics is better for separating viable kidney cancer CTCs with high purity without losing integrity for subsequent molecular analysis.

Microfluidic tools like genomic, transcriptomic, and proteomic profiling allow for single-cell analysis of CTCs. Single-cell sequencing within microfluidic tools has also been used to demonstrate kidney cancer CTC population heterogeneity, which is critical to metastatic potential and therapeutic resistance mechanisms. Microfluidic tools, in combination with dielectrophoretic sorting and culturing in microchambers, permit the functional characterizations of CTCs, including proliferation and drug sensitivity profiles (141). Probing individual CTCs gives more knowledge about the growth of tumors, enabling better attack with new therapies.

#### 5.2.2 Detection of exosomes and extracellular vesicles

Exosomes and other EVs are nanoscale tumor cell-derived vesicles at the center of tumor development, immune regulation, and intercellular communication. Vesicles contain molecular cargo in terms of protein, RNA, and lipids, reflecting the tumor’s physiological state (30). Kidney cancer exosome detection and characterization using miniaturized, high-throughput, label-free, and low-volume methods are far more effective using microfluidic technologies than conventional ultracentrifugation-based methods. Microfluid-based exosome isolation methods include immunoaffinity capturing, size-exclusion filtering, and acoustic-based separation. Immunoaffinity-based microfluidic devices use antibody-functionalized surfaces against exosomal markers CD9, CD63, and CD81 for selective enrichment and isolation (146). Using a predefined set of surface markers, although performance is high, renders the approach somewhat limited (146).

Size-based filtration and microfluidic deterministic lateral displacement techniques allow size- and deformability-dependent separation of EVs independent of molecular markers. Acoustic-microfluidic devices employ ultrasonic waves to interact with vesicles, enabling label-free, high-throughput exosome separation (147). Microfluidic integration with biosensors has also provided real-time exosome analysis, an application for tracking kidney cancer development and drug treatment. SPR-based microfluidic devices allow direct protein analysis of exosomes, while microfluidic digital PCR and sequencing based on droplets analyze exosomal RNA and DNA mutations. Such methods have rendered exosomal biomarkers applicable to non-invasive liquid biopsies for kidney cancer diagnosis (30).

#### 5.2.3 Microfluidic platforms for drug screening and resistance testing

Microfluidic devices are engineered as effective tools for drug screening and assessment of drug resistance in kidney cancer. Conventional drug screening models, such as 2D cell culture and animal models, do not accurately mimic the tumor microenvironment. Hence, there are differences in drug efficacy and mechanisms of drug resistance. Microfluidic organ-on-a-chip models overcame this limitation by mimicking physiologically relevant microenvironments, which allow for the monitoring of drug responses in real-time at a microscale. Microfluidic platforms for drug screening against cancer are commonly made up of patient-derived tumor spheroids or cells in perfusable microchannels that replicate *in-vivo* environments. They support controllable delivery of chemotherapeutic, targeted, and immunotherapeutic agents with measurement of the impact on cell viability, cell growth, and apoptosis. Tong et al. present an application of kidney cancer chip models for demonstrating patient-specific responsiveness versus TKIs and immune checkpoint inhibitors, showing the potential for developing precision oncology therapies (148).

Microfluidics is utilized to explore drug resistance mechanisms via real-time observation of molecular adjustments of cancer cells. Kidney cancer develops drug resistance against TKIs and mTOR inhibitors through genetic mutations and activation of alternate pathways (149). Microfluidic chips coupled with single-cell RNA sequencing and CRISPR-based functional genomics have shown resistance-related gene signatures and combinatorial effective therapeutic approaches for reversing drug resistance. Microfluidic co-culture models of tumor cell-immune cell-stroma interaction have been established. The models elucidate processes by which the tumor microenvironment promotes drug resistance and immune evasion. Microfluidic co-culture models of tumor-infiltrating lymphocytes and kidney cancer cells demonstrate heterogeneous immune checkpoint inhibitor responses, which inform mechanisms of resistance to immunotherapy.

#### 5.2.4 Current detection limits and analytical performance of microfluidic platforms

The promising applications of microfluidics for liquid biopsy in RCC must be weighed against current analytical performance and practical detection limits. For CTCs, label-free microfluidic enrichment devices have demonstrated detection of spiked inputs on the order of ~20 cells per milliliter of whole blood in non-RCC oncology models (150). This should be treated only as a generic benchmark: RCC-specific, clinically validated CTC microfluidic platforms remain under active development, and performance is often reported as capture efficiency/yield rather than a formal LOD. For ctDNA, droplet-based digital PCR (ddPCR) on microfluidic platforms achieves analytical variant-allele-fraction (VAF) limits around 0.1%-0.01% (28, 123). In RCC, however, clinical sensitivity is frequently constrained not by platform LOD but by biologically low ctDNA shedding, leading to low plasma tumor fraction in many patients (125). Thus, while microfluidic technologies continue to push analytical limits downward, their real-world utility in RCC must be interpreted considering disease-specific biomarker biology (e.g., low ctDNA abundance, EMT-related antigen loss for CTCs) and may benefit from tumor-guided panels and methylation/fragmentomic enrichment strategies.

### 5.3 Microfluidic devices for kidney cancer biomarker detection

Microfluidic devices are a perfect platform for sensing biomarkers for kidney cancer, given that they are sensitive, utilize less sample volume, and are faster. They support real-time detection for a wide range of biomarkers.

#### 5.3.1 Design and fabrication of microfluidic devices

The microfluidic device design and fabrication include microchannel geometry, surface modification, and biomarker-specific capturing processes. Microfluidic devices are often fabricated using polydimethylsiloxane, glass, or thermoplastics. This varies depending on the benefits of biocompatibility, optical clarity, and simplified processing. With high fluid dynamics and biomarker interaction control, soft lithography, photolithography, and 3D printing have emerged as common methods for fabricating microfluidic chips. The use of microstructures with specific functions allows these devices to facilitate biomarker detection and separation. Deterministic lateral displacement and inertial microfluidic channels work by using the different sizes and shapes between blood cells and CTCs to separate them. The use of nanoporous membranes on microfluidic chips helps reliably extract more purified and recoverable exosomes and EVs (151).

Markers for kidney cancer can be more accurately detected when surfaces are treated with antibodies, aptamers, or nanomaterials. Surfaces made from plasmon resonance (SPR), electrical sensors, and instruments for spotting fluorescence help power this type of microanalysis. Being able to communicate, platforms are able to spot biomarkers fast and avoid pursuing many prolonged steps. Using microfluidic devices has reduced and automated the analysis of biomarkers, assisting in better decisions about kidney cancer in healthcare (152).

#### 5.3.2 Case studies of microfluidic devices for kidney cancer detection

Microfluidic chips help doctors find kidney cancer biomarkers quickly, making it easier to identify the disease early and manage future care. Highly efficient CTC separation from RCC patients is achieved on inertial microfluidic chips without requiring labels. Using microfluidics lets researchers detect gene alterations related to drug resistance more easily and proves the technique’s medical benefits. In a different approach, microfluidic devices have also been designed to isolate RCC exosomes selectively by using immunoaffinity selection combined with a nano-sized filter (153). CAIX is an option to focus on treatment because it becomes more active in ccRCC. Nevertheless, CAR T cell therapy targeting CAIX has often resulted in a greater risk of on-target off-tumor toxicity in the past. As a response, Wang Y. et al. changed the CAR-T cells to target tumor cells expressing a high amount of CAIX and ignore healthy areas, such as the bile duct, that have very low CAIX (111). Because this approach improves outcomes without major side effects, CAIX can be considered a suitable and safe target for RCC therapy with CAR-T cells.

A comparison between exosomal RNA levels and those seen in tumor tissue suggests exosomal RNA might serve as a biomarker for tracking disease development in RCC. Microfluidics are being used more often to look for ctDNA mutations in blood without needing surgery. Microfluidic chips that incorporate multiple reaction channels, like digital PCR, can uncover changes in the VHL gene from a tiny sample of plasma with great sensitivity, as investigated by Sumiyoshi et al. (154). Compared to normal biopsies, this method allows doctors to see tumor growth in real-time and evaluate therapy effectiveness. Profiling molecular changes as they happen through microfluidics is a key reason why microfluidic platforms are becoming important for personalized medicine in RCC.

#### 5.3.4 Comparison with conventional diagnostic methods

Conventional diagnostic techniques for kidney cancer, including imaging studies and histopathology, are useful for tumor characterization and staging. Conventional approaches, however, are limited by what they cannot do, with a key limitation being what they cannot detect, namely, minimal residual disease, invasiveness of a biopsy, and delayed provision of molecular data. Microfluidic devices overcome the above limitation by allowing real-time, non-invasive detection of biomarkers with greater sensitivity and specificity. Conventional tissue biopsy is still considered the gold standard for making a definitive diagnosis of kidney cancer (108). The approach, however, is risky, with complications including bleeding, sampling errors, and insufficiency of tumor heterogeneity representation. Microfluidic-based liquid biopsies, on the other hand, enable continuous monitoring of the progression of cancer based on blood-based biomarkers without invasive treatments. Capture and detection of CTCs, exosomes, and ctDNA at multiple time points improve monitoring of treatments and assessment of prognosis.

Conventional biomarker detection methods, such as mass spectrometry, require large quantities of samples and are time-consuming. The use of microfluidics evades the shortcoming as the miniaturized chambers and automated assay protocols help reduce assay time and reagent volume (27). Microfluidics is also incorporated into biosensing technologies, which enhance analytical sensitivity. This enables the detection of low-abundance markers that conventional assays would be likely to miss. There are, however, issues with the standardization of microfluidic assays, approval from regulating agencies, and insufficient studies to establish reproducibility and utility in clinical environments.

### 5.4 Point-of-care microfluidic diagnostics

Point-of-care microfluidic diagnostics are important instruments in kidney cancer diagnosis and follow-up at an early stage. Low-cost, portable instruments enable speedy, specific, and sensitive analysis of biomarkers, which is possible even without advanced lab equipment. The handheld POC device miniaturizes reagent consumption and achieves high-throughput screening and hence is highly suitable in scarce-resource environments (155). This section assesses the current prospects using handheld microfluidic tools, smartphone-enabled integration, and utility in environments where there is poor healthcare support.

#### 5.4.1 Development of portable and cost-effective microfluidic devices

Microfluidic diagnosis devices made for portable use attain a balance between ease of use, sensitivity, and cost. Standard diagnosis for kidney cancer, for instance, is expensive and requires specialized instruments, including imaging and histopathology. Microfluidic devices at the point of care provide a distributed platform wherein on-demand testing of relevant biomarkers, including CTCs, exosomes, and ctDNA, is feasible. Paper-based microfluidic devices rely on hydrophilic channels patterned on cellulose or nitrocellulose substrates for fluid control (145). This does away with external pumps and sophisticated instrumentation. A colorimetric or fluorescence-based detection gives a rapid, cheap biomarker quantitation, which makes them probable for use at the point of care.

The development of thermoplastic microfluidic chips represents a big advance toward portable diagnostic device fabrication. The chips demonstrate greater optical clarity, chemical integrity, and scalability. However, it is difficult for handheld devices to match lab-based assay sensitivity and specificity. Coupling electrochemical and surface plasmon resonance biosensors permits sensitive detection of early-stage kidney cancer biomarkers (156).

#### 5.4.2 Integration with smartphone-based technologies

Smartphone-based microfluidic diagnostic devices are a major innovation in POC testing. The universal use of smartphones with high-resolution cameras, processing capabilities, and wireless connectivity has made it feasible for mobile health (mHealth) technology to detect kidney cancer. These devices enable real-time acquisition and analysis of data and discussion remotely, eliminating distance between the clinician and patient. Several smartphone-based microfluidic platforms for cancer diagnosis have been reported. Smartphone-based image processing software has been integrated with colorimetric microfluidic assays for the quantification of biomarkers for kidney cancer (157). The assay output is recorded using a smartphone camera, and specially designed mobile apps measure color difference contrast for a quantitative output. The process does away with costly laboratory facilities and makes it possible for untrained non-professionals to conduct diagnostic testing.

Innovations in the line of smartphone-integrated microfluidics involve integrating portable readers of fluorescence into the devices. Fluorescent biosensors increase the sensitivity of microfluidic assays based on biomarker-specific fluorescence signals. Using technology alongside smartphones ensures signal acquisition and transmits data for cloud analysis. The new technology has been used in CTC and exosome RNA analysis for patients affected by kidney cancer. The successful use of diagnosing kidney cancer illustrates a useful application in liquid biopsies that do not require invasive procedures. The application of smartphone-based microfluidic platforms is integrable with cloud-based computation and AI platforms to support analysis of data (158). The application of machine learning algorithms ensures discrimination against kidney cancer subtypes. The analysis examines the varied occurrence of biomarker expressions from the analysis of microfluidic assays. The application of AI-based analysis enhances diagnostic outcomes by increasing diagnostic accuracy through real-time personalized risk assessment. Notably, such an application increases the scope for personalized medicine to treat kidney cancer.

#### 5.4.3 Potential in resource-constrained settings

In resource-scarce settings, access to microfluidics is impacted by economic and infrastructure-related issues. Point-of-care microfluidic diagnosis is a realistic substitute that provides low-cost, rapid, and precise detection of biomarkers in a low-resource clinical setting. The POC microfluidic devices are best suited and offer ease of use and portability for community-based screening, for instance, in the high prevalence of kidney cancer. This offers a major strength of having low reliance on peripheral hardware due to its portability. Most platforms function using capillary forces or passive flow, which eliminates the need for power-intense pumps and controllers. Battery-driven or solar-driven microfluidics facilitate functionality if there is no guarantee of electricity (159). Point-of-care microfluidic diagnostics allow real-time diagnosis of disease, which lessens patient visits to distant health centers (155). This is useful for targeted immunotherapy for kidney cancer patients. In such cases, frequent monitoring is crucial for monitoring the response to the drug and the progression of the disease. The devices employ on-site biomarker analysis to enhance patient adherence and allow timely therapy adjustment.

In resource-scarce backgrounds, POC microfluidic diagnostic platforms are subject to considerations of affordability and sustainability. Cost-effective microfluidic fabrication processing through processing and screen printing is used to produce inexpensive, one-use-only microfluidic strip tests at a reduced cost compared to conventional lab tests (160). Efficient innovations facilitate the easier rollout of such platforms to underserved areas. The most significant issue with the implementation of POC is the lack of standardization of assay performance across different populations and environments. The assay performance is susceptible to inconsistencies in the composition of the sample, temperature, and humidity, necessitating rigorous quality control. Clinical validation and approval facilitate the integration of microfluidic POC devices into national health schemes (161).

## 6 Integration of microfluidics and biomolecular techniques

### 6.1 Combined approaches for enhanced detection

The integration of microfluidic techniques and molecular diagnostics signifies a crucial progress for precision oncology in RCC. These hybrid systems facilitate quick, high-throughput, and minimally invasive identification of circulating biomarkers—such as CTCs, ctDNA, and exosomes—by combining micro-scale fluid control with sophisticated biomolecular tests, all while maintaining sample integrity for subsequent omics studies. This method enables immediate tumor observation, prompt identification, and personalized treatment enhancement (28, 29).

#### 6.1.1 Microfluidics and biomolecular techniques usage

Connecting microfluidic systems to PCR, ELISA, and NGS has greatly improved the finding of biomarkers linked with both viral and cancer-related illnesses. With this mix, data is collected fast, errors can be found early, and the system becomes more sensitive. In contrast to previous techniques, using microfluidics, biosensors, aptamer-based assays, and lab-on-a-chip systems helps simplify views on biomarkers and aids in the diagnosis of kidney cancer, as shown by Liu et al. (152). The team discovered that it was possible to correctly identify and isolate individual CTCs from the sample using SERS incorporated into microfluidic systems. In addition, Bakhshi et al. found that test results from aptamer-nanoparticle-based reading on microfluidic devices reached high diagnostic accuracy and sensitivity rates of 95–100% in cancer detection (162). Abu-Dawas et al. pointed out that lab-on-a-chip devices help detect biomarkers and make it possible for people to get tested at the point of care (142).

In addition, Liu et al. revealed that liquid biopsies based on microfluidic devices are able to find squamous cell carcinoma at an early stage and use 30% fewer tissue biopsies compared to usual methods (152). Hawsawi et al. also report that microfluidics helped distinguish breast cancer accurately and resulted in a considerable amount of data helpful for designing treatment plans for RCC (163). Also, Shapiro et al. have evaluated the use of microfluidics to track how kidney cancer responds to treatment in real time, significantly increasing the benefits of this therapy (164). Such literature illustrates evidence supporting the revolutionary nature of microfluidics in kidney cancer diagnosis and treatment.

#### 6.1.2 Case studies on integrated platforms for kidney cancer diagnosis

Numerous RCC-centered investigations illustrate the translational promise of cohesive microfluidic–molecular systems. Miller et al. (2018) created a microfluidic ccRCC-on-a-chip to study tumor-induced angiogenesis using primary patient-derived ccRCC cultures and demonstrated pharmacological blockade of angiogenic sprouting (165). In a similar manner, Sumiyoshi et al. (2021) employed a droplet-based PCR microchip to identify VHL and PBRM1 mutations in plasma ctDNA from RCC patients, facilitating dynamic treatment monitoring and non-invasive molecular profiling (154). In a different method, Lu et al. (2021) created a microchip for exosome isolation that incorporates artificial intelligence to recognize protein signals for tumor detection (166). In addition, a system based on CRISPR-Cas12a is presently being tested to diagnose kidney cancer through the study of exosomal RNA. Wang M. et al. reported that the system made the correct distinction between cancer and normal cells more than 90% of the time, explaining its use in cancer detection (167). The system has been tested to work with just 10 femtomoles, a sensitivity higher than regular PCR. Microfluidic chips can work with graphene oxide-based biosensors to greatly improve the sensitivity and label-free detection of VEGF, which is significant for RCC. With this system, estimating biomarkers becomes more efficient and requires less time. De Araujo et al. have also shown that applying silica-core@dual quantum dot-shell nanocomposites in LFIAs brought about 95.54% accuracy and required only 15 minutes (168). Micofluidic-ddPCR, together with nanoparticle amplification of fluorescence, is now being used to detect ctDNA in kidney cancer cases. Stutzmann et al. also studied the use of microfluidics in mass spectrometry-based proteomics, discovering a highly sensitive approach to researching conditions like ccRCC and offering many insights into tools like the PeptiCHIP method (169). The examples demonstrate that integrating microfluidic and biomolecular devices is useful for kidney cancer testing. Modern technology is making it easier to identify and treat kidney cancer using faster, more accurate, and less harmful processes.

#### 6.1.3 Challenges in scaling up and commercializing integrated technologies

Making progress in medicine is still difficult with microfluidic-biomolecular integrated platforms. It is important to care about reproducibility because it is still not possible to have the same results with every batch. Among the things that need control in microfluidics are the dimensions of channels, changes in surface chemistry, and the functional stability of any chemicals used (170). Some of the main challenges in the field have been solved with photolithography, soft lithography, and 3D printing, but there is still some uncertainty that keeps technology from being accepted in clinics. Regional approval by the FDA is also a major point of concern due to the need to compare traditional animal results with *in vitro* data (171). The FDA and EMA require strong validation tests to prove that medicine is reliable, effective, and safe, given the strict validation of protocols.

Microfluidic diagnostics have not been widely adopted in healthcare because there are no well-defined standard protocols for their use (172). Microfluidic devices have their fluid dynamics that, unlike regular systems, require new and unique validation methods that scientists are still studying. Adopting these technologies in medical care requires conference-wide guidelines made by regulatory authorities to govern their use. Notably, the money needed to develop and build microfluidic devices still deters many since the costs can cancel out the benefits of faster analyses and less-used reagents (173). Switching from the early stage to mass production requires methods such as injection molding and high-throughput processing to become economically successful.

Making sure that microfluidic medical devices are both safe and convenient for use is challenging. The architecture of the device must include special filters to avoid reactions between biological samples and the chip surfaces. In addition, linking automated tools with simple user interfaces makes it possible to introduce these advanced diagnostics in hospitals without difficulty. Implementing advanced diagnostics in medical care is slow without thinking about user-centered design. As stated by Birhanu, important steps forward in the field rely on teamwork between leading businesses, regulatory groups, and scientific authorities (174). Standardized manufacturing, improved guidelines, and more automation will help new laboratory advancements be used in medicine more quickly and effectively. As the main challenges are solved, microfluidic-biomolecular platforms will likely have a greater impact on kidney cancer diagnosis and personalized medicine.

### 6.2 Role of artificial intelligence in microfluidics and biomolecular analysis

AI improves microfluidics diagnostics by interpreting intricate multidimensional datasets produced from microfluidic analyses. Machines trained using AI, such as deep learning, can analyze exosome images for tumor marker detection (166). A convolutional neural network applied to contrast-enhanced CT images effectively distinguished between benign and malignant renal tumors, achieving an AUC of 0.918 (175) A separate study connected microfluidic mass spectrometry with AI feature selection to pinpoint lipidomic biomarkers related to unfavorable outcomes in metastatic RCC (176). The combination of AI pipelines with datasets produced by microfluidics speeds up biomarker identification, enables automated evaluation, and enhances the predictive power of molecular diagnostics in managing RCC.

#### 6.2.1 Machine learning algorithms for data analysis

Machine learning techniques, including deep learning, support vector machines (SVM), and convolutional neural networks (CNN), are critical in analyzing large data sets from microfluidic and biomolecular platforms. The machine learning techniques detect faint patterns from high-dimensional data, improving early kidney cancer diagnosis. AI-powered microfluidic platforms analyze CTCs and EVs with greater precision than conventional methods. Reinforcement learning enhances the feature selection and classification process and increases diagnostic performance and patient outcome predictions.

Current studies have shown the efficacy of microfluidic diagnosis through machine learning compared to the conventional system through better fluid flow dynamics prediction (177). The study by Park, Kim & Jeon affirms that diagnostic accuracy represents breakthroughs in these ML-based devices, enabling better droplet management, automating microfluidic channel design, and real-time biological sample analysis (177). In addition, deep learning-based single-cell transcriptomes on microfluidic assay from a study by Ge et al. revealed new insights into potentially individualized therapy through detailed tissue maps using high-dimensional complex data that enhances better prediction and molecular modeling (178).

Biomedical scientists are relying more on AI microfluidic technologies to diagnose diseases more accurately and simplify time-consuming experiments. According to Asadian et al., microfluidics provides many positive aspects, but the large, complex datasets it creates are hard for humans to deal with (179). Thanks to the use of deep neural networks and other algorithms in artificial intelligence, all these difficulties are now being handled automatically. Reviewed applications apply AI to detect diseases and group cells and help with diagnostics at the bedside as this technology speeds, improves, and simplifies the process without requiring expert staff. These advances highlight the revolutionary potential that AI can have for kidney cancer diagnosis based on microfluidics. As machine learning algorithms become increasingly sophisticated with time and combine with the analysis of biomolecules, the future of non-invasive, highly accurate detection and monitoring of kidney cancer looks brighter.

#### 6.2.2 AI-driven biomarker discovery and validation

With AI, important biomarkers are discovered sooner and in greater detail through genomic, proteomic, and metabolomic studies of kidney cancer. Routine NLP checks a large body of science-related information for likely significant biomarkers. Additionally, machines are able to analyze huge amounts of multi-omics data to find the connections among biomarkers, which cannot be done by standard analysis. New data imply that discovering biomarkers with AI enhances their accuracy in both medical diagnoses and forecasting outcomes (180, 181). Reports from Azuaje et al. show that analyzing kidney cancer patients’ information at various levels with machine learning improved the results of treatment, how long patients survive, and the chance of finding kidney cancer early (180). It was also mentioned by Tran et al. that by blending multi-omics analysis and AI, survival prediction in patients was better because it helped cover all kinds of assessment data and made sure models were properly validated (181). Overall, this evidence reveals that using both machine learning and deep learning helps microfluidic platforms much better assess biomarkers.

Using AI in microfluidic testing helps find and confirm the presence of new biomarkers more easily. Screening data quickly and accurately becomes possible when many samples are used, and the process is automated. As shown by Serrano et al., the use of AI has greatly improved microfluidic screens by reducing validation time and supporting both personalized medicine and target improvement (172). Based on their meta-analysis, the authors found that using AI tools helped standardize the discovery of biomarkers by reducing differences between various research teams by around 93.7%. Using AI, microfluidics is assisting oncology by enabling the identification and verification of important biomarkers. Also, with advances in computer platforms, doctors can predict more accurately useful biomarkers in kidney cancer, which will simplify and personalize the treatment and diagnosis process.

#### 6.2.3 Prospects of AI in kidney cancer research

Upgrading microfluidic setups with AI looks promising for speeding up and cutting the financial costs of diagnosing kidney cancer, as well as detecting other illnesses. AI-assisted continuous monitoring of important biomarkers allows clinicians to quickly change a patient’s treatment plan when there are changes in their condition. Wang C. et al. revealed that relying on AI and ctDNA or EVs from biofluids improved both how swiftly and accurately early-stage tumors were detected (182). Point-of-care tests improve significantly if AI advances, and these techniques are used in handheld devices. McGough et al. have observed that using AI and mobile devices allows anyone to carry out rapid kidney cancer detection with very limited need for centralized laboratory equipment (183). Similarly, introducing machine learning into sample analysis quickens and improves the accuracy of the diagnostic processes, which is good for both research results and how care is delivered.

New AI technologies, such as federated learning, will also improve data protection and predictive capabilities by training the models on decentralized hospital networks without violating patient confidentiality. Shehata et al. demonstrated that AI-based diagnostic studies report a yield of 82% to 91% accuracy range regarding differentiation of both malignant and benign renal tumors compared with the conventional diagnostic approaches (184). AI-based microfluidic precision oncology will enable highly personalized treatment regimens. Predictive analytics from deep learning models will be utilized for real-time prediction of treatment effectiveness, and clinicians can make data-driven regimen changes. Digital microfluidic drug screening reduces side effects and improves the response of cancer patients via precision medicine, which is better performed through on-chip screening (185). As AI technology advances, diagnosis and treatment of kidney cancer will increasingly be guided by innovations with earlier and more precise interventions and customized therapeutic approaches that achieve the best possible patient outcomes.

## 7 Challenges in RCC diagnostics

RCC presents a unique and interrelated spectrum of diagnostic challenges that extend beyond the general liquid biopsy framework (**Table 2**). Technically, current microfluidic platforms for CTCs and EVs still suffer from EpCAM-dependent capture bias, clogging under realistic hematocrit conditions, shear-induced cell loss, matrix effects such as hemolysis and high viscosity, and lot-to-lot variability of functional ligands. On-chip cell expansion and consistent transfer to downstream omics workflows remain limited to specialized laboratories, while cost-effective scaling through robust thermoplastic manufacturing is still incomplete (140, 141, 151, 173). Molecularly, the typically low fraction of ctDNA in RCC amplifies pre-analytical and library preparation bias, necessitating the use of unique molecular identifiers (UMIs) for error suppression, tumor-informed gene panels focusing on VHL, PBRM1, BAP1, and SETD2, and complementary methylation or fragmentomic assays. Urine-based workflows, optimized for selected biomarkers, may even outperform plasma in certain contexts (28, 124, 125). Biologically, epithelial-mesenchymal transition (EMT) and phenotypic plasticity reduce affinity-capture yields, EV populations display high heterogeneity, and the VHL-hypoxia pathway drives extensive spatial and temporal tumor heterogeneity. Not all lesions actively shed, and papillary and chromophobe subtypes differ in their shedding behavior. These factors support serial plasma-plus-urine sampling, RCC-specific surface targets such as CAIX or CD147, and tumor microenvironment (TME)-on-chip systems to contextualize biomarker function and drug response (116, 117, 144). Regulatory and validation gaps persist due to the lack of RCC-specific analytical reference materials, few multicenter prospective studies with blinded endpoints, and absence of standardized thresholds for minimal residual disease (MRD) or longitudinal monitoring. Alignment with STARD reporting guidelines, European Association of Urology (EAU) recommendations, and laboratory quality frameworks (CLSI/ISO 15189), together with participation in external quality assessment schemes, is needed to improve reproducibility and regulatory readiness (185). Economically, custom microfabrication, specialized operator training, and the supply chain for capture ligands maintain high per-test costs. Furthermore, ultra–low VAF sequencing and bioinformatics pipelines face reimbursement and cost-effectiveness uncertainty. Potential solutions include injection-molded or printable chips, smartphone- or edge-AI-based readers, and formal health-economic comparisons with evolving imaging and biopsy pathways (27, 158, 173). Data and AI challenges add further complexity: image and signal formats from microfluidic devices lack standardization, labeled RCC datasets remain scarce compared to other cancers such as lung or breast, and model generalization is hindered by device and domain variability. Developing benchmark RCC datasets, AI-assisted quality control and variant calling, and privacy-preserving data-sharing frameworks (e.g., federated learning) could enhance analytical robustness and reproducibility (179, 183). Finally, in clinical translation, sample routing and chain-of-custody from chip to single-cell RNA sequencing, spatial profiling, or organoid-based drug screening are rarely seamless. Single-cell and spatial omics remain costly and time-consuming. Establishing pre-specified standard operating procedures (SOPs) for chip-to-omics workflows, integrating organoid and single-cell data, ensuring CLIA/IVDR compliance, and reporting turnaround time and clinical actionability are essential steps for incorporating these diagnostic tools into multidisciplinary tumor boards and longitudinal RCC care (164, 186).

**Table 2:** Unresolved challenges in RCC-specific microfluidic CTC capture.

Challenge	RCC-specific issue	Microfluidic impact/gaps	Next steps	Ref.
Low/heterogeneous epithelial markers	RCC CTCs often downregulate EpCAM/cytokeratins via EMT or non-epithelial phenotypes → affinity chips miss cells.	Anti-EpCAM devices under-recover; single-marker strategies underperform; RCC label-free routes underused.	RCC-tailored panels (e.g., CAIX, CD147) and EMT-aware cocktails; modular chips to swap ligands rapidly.	(28, 65, 141)
Physical similarity to blood cells	RCC CTCs can be deformable and size-overlapping with leukocytes.	Pure size/deformability filters lose specificity or clog at realistic hematocrits.	Hybrid cascades (inertial + DLD + acoustic) with clog-resistant layouts; RBC/platelet pre-processing; validated label-free design rules/sims.	(140, 141)
Rarity at baseline	RCC is relatively “low-shedding” vs breast/lung; counts frequently low/undetectable.	Throughput–sensitivity trade-off; need high-volume equivalent processing without cell stress.	High-throughput inertial/Dean-flow; channel parallelization; gentle recirculation preserving viability.	(28, 125)
Sample pre-analytics	Tube type, delays, agitation, anticoagulant can damage fragile CTCs.	Recovery drops under routine phlebotomy/transport; on-chip viability for omics suffers.	RCC-specific pre-analytical SOPs; stabilizing tubes; immediate on-chip fixation/encapsulation.	(28, 125, 126)
On-chip viability & downstream assays	Viable CTCs needed for culture, drug testing, multi-omics.	Shear, surface chemistry, elution conditions impair viability/yield.	Low-shear surfaces; biocompatible coatings; gentle release chemistries; integrate single-cell arrays/organoid seeding.	(26, 170, 171)
Standardization & clinical validation	Few RCC-specific CTC benchmarks; heterogeneous endpoints.	Hard to compare devices/claims; limited prospective RCC cohorts.	RCC-tailored reference panels; multi-centre, blinded studies aligned to clinical endpoints.	(28, 65)
Manufacturability & cost	Complex 3D fluidics/multi-physics actuation raise costs.	Limits disposables and routine clinical use.	Low-cost fab (e.g., MINX), μPAD modules, smartphone/AI readers for fieldable analytics (note: general microfluidics evidence).	(145, 157, 158, 160)
Integration with TME biology	RCC TME is hypoxic/angiogenic with heterogeneous clonal ecology.	Single end-point capture misses functional heterogeneity and dynamics.	Pair CTC capture with TME-on-a-chip co-culture; longitudinal phenotyping of captured cells.	(144)

EMT, epithelial–mesenchymal transition; DLD, deterministic lateral displacement; μPAD, microfluidic paper-based analytical device; TME, tumor microenvironment.

## 8 Conclusion

RCC remains a significant global health challenge, with high mortality rates largely due to late diagnosis and therapeutic resistance. While traditional diagnostic and treatment approaches have improved outcomes, they are insufficient to address the disease’s complexity and heterogeneity. The integration of microfluidics and molecular diagnostics marks a transformative step toward non-invasive, real-time, and highly sensitive detection of RCC biomarkers such as CTC, ctDNA, and exosomes. Coupled with advancements in single-cell analysis, next-generation sequencing, and multi-omics integration, these technologies offer unprecedented opportunities for personalized and precision oncology. Despite promising developments, challenges remain in standardization, clinical validation, and large-scale implementation. Ongoing research and interdisciplinary collaboration are essential to translate these innovations into routine clinical practice and ultimately improve prognosis and quality of life for patients with kidney cancer.
